# Computational surveillance of Colombian public pharmaceutical procurement using public administrative data: A reproducible analysis of a closed 2020–2025 cohort

**DOI:** 10.1371/journal.pone.0350967

**Published:** 2026-09-11

**Authors:** Andrés Soto

**Affiliations:** Independent Researcher, Bogota, Colombia; Public Library of Science, UNITED KINGDOM OF GREAT BRITAIN AND NORTHERN IRELAND

## Abstract

**Introduction:**

BigLoI monitors Colombian public pharmaceutical procurement from 2015 onward. For this manuscript, the source cohort comprised 162,271 candidate pharmaceutical contracts from 2020 to 2025. After contract-level review excluded 441 records explicitly concerning veterinary, animal-health, or agricultural use, the corrected closed analytical cohort comprised 161,830 contracts.

**Objective:**

To describe the design, implementation, and findings of a reproducible computational infrastructure for surveillance of Colombian public pharmaceutical procurement.

**Methods:**

Public APIs from SECOP-II, INVIMA, and SISMED were integrated into a reproducible architecture combining PostgreSQL, statistical analysis, and a public-facing observatory. Candidate records whose supplier names indicated possible veterinary or agricultural activity underwent contract-object review; 441 records with explicit animal, veterinary, or agricultural scope were excluded, while human-health and ambiguous records were retained conservatively. A Z-score engine was implemented to flag contracts with atypical total values within therapeutic categories. As a clearly secondary technical-feasibility module, smart-contract automation of a payment workflow was tested on the Sepolia testnet only to verify predefined digital state transitions under simulated conditions. The corrected closed analytical cohort included 161,830 pharmaceutical contracts from 2020 to 2025, while post-2025 records remained available only for live platform monitoring. Monetary results were reported primarily in COP; secondary USD equivalents were included only as approximate interpretive references, with detailed conversions relegated to S1 Table.

**Results:**

Among 146,594 contracts analyzed in categories with at least 10 observations, 664 contracts (0.45%) triggered a statistical alert with absolute Z-score greater than or equal to 1.5 sigma. The alert rate among Z-score-eligible contracts increased from 0.30% in 2021 to 1.31% in 2025. Antibiotics showed a category-level maximum Z-score of 8.89. The top 3% of suppliers concentrated 85.8% of total contracted value. As a clearly secondary module, the Sepolia smart-contract prototype confirmed only that predefined digital state transitions could be executed under simulated testnet conditions; it provides no evidence of real-world payment-cycle reduction, realized savings, or institutional deployability.

**Conclusions:**

A reproducible national-scale computational infrastructure identified atypical procurement patterns and documented marked market concentration in Colombian public pharmaceutical procurement. These descriptive findings may inform auditing, public health policy discussions, and health data governance, but they do not by themselves establish wrongdoing or prescribe specific reforms. Statistical alerts remain exploratory prioritization signals rather than evidence of corruption or fraud. The blockchain module should be interpreted strictly as a complementary technical proof of concept tested under simulated conditions and not as operational evidence on real-world payment performance, savings, or implementation readiness.

## 1 Introduction

Public procurement of medicines in Colombia is monitored in BigLoI through a broad SECOP-II universe that now exceeds 272,000 contracts from 2015 onward. For the purposes of this manuscript, the corrected closed analytical cohort corresponds to 161,830 pharmaceutical contracts from 2020 to 2025 after contract-level veterinary exclusions. Despite this scale, the system lacks reproducible real-time surveillance tools to detect atypical procurement patterns, market concentration, or potential supply risks.

The World Health Organization has estimated that 10% to 25% of global health expenditure is lost to corruption, inefficiency, and administrative errors [1]. In Colombia, official audits have documented high-cost medicine overpricing, prolonged payment delays, and recurring episodes of shortages [2–4].

Integration of public APIs makes it possible to build procurement-surveillance platforms without access to confidential information. Z-score analysis is a practical approach for detecting atypical patterns in large procurement datasets [5], while smart contracts and semantic retrieval expand the range of possible automation and evidence-access tools [6,7]. Initiatives such as ProZorro, OCDS, and OLAF illustrate the feasibility of these approaches in public-procurement transparency [8–10].

BigLoI (Business Intelligence for Government Logistics and Operations Intelligence) was developed to address this gap. This article documents its methodology and findings as a contribution to the debate on computational tools for governance of the Colombian health system.

## 2 Methods

### 2.1 Study design

This was a technological-development study with longitudinal observational analysis of public procurement data. Only openly accessible Colombian public data sources were used. No clinical data or patient-level personal data were included.

**Ethics statement.** This study used only publicly accessible administrative procurement records published by Colombian open-data sources (SECOP-II via datos.gov.co, INVIMA, and SISMED). It did not involve human participants, patient-level or clinical data, identifiable private information, biological samples, recruitment, intervention, or any contact with individuals. Accordingly, the study does not constitute human-subjects research and did not require review or approval by an institutional review board or research ethics committee. Under Colombian Resolution 8430 of 1993, analysis of public, non-identifiable administrative records corresponds to research without risk and falls outside the scope of human-subjects ethics review. Informed consent was not applicable because the study did not involve human participants or identifiable personal data. All analyzed data are publicly available and were used in aggregate for the surveillance of public procurement.

### 2.2 Data sources

**SECOP-II (Electronic Public Procurement System).** The public Socrata API from datos.gov.co was used for automated download of pharmaceutical procurement contracts. Initial inclusion required the presence of at least one of 19 pharmaceutical terms in the contract-object description (S1 Appendix). This keyword strategy is a high-recall screen rather than a gold-standard classifier: it can generate false positives (non-pharmaceutical or animal-health contracts that share lexical terms) and false negatives (human pharmaceutical contracts whose objects omit the inclusion vocabulary). Because terms such as vaccine and antibiotic can also describe animal-health procurement, supplier names were screened for veterinary or agricultural indicators and 519 candidate records underwent contract-object review. Records were excluded only when the object explicitly indicated veterinary, animal-health, zoonotic intervention on animals, or agricultural use outside the human-pharmaceutical scope. This review excluded 441 records, retained 27 records with explicit human-health or lexically incidental scope, and conservatively retained 51 ambiguous records. The review decisions and source-row identifiers are included in the reproducibility package. No independently adjudicated external reference set was available to estimate sensitivity or specificity of the full keyword taxonomy; the contract-level review therefore serves as targeted quality control for a known contamination pathway rather than as complete taxonomy validation. The platform monitoring window extends from January 2015 onward, but the closed analytical cohort used in this manuscript was restricted to complete calendar years 2020–2025.

**Recovered local PostgreSQL source used to reproduce the manuscript cohort:** 956,157 total SECOP-II records; 165,549 candidate pharmaceutical records including live 2026 monitoring rows; 162,271 candidate contracts in the source 2020–2025 cohort; 161,830 contracts after contract-level veterinary exclusions.

**SECOP-II monitored universe available to the platform:** 272,814 unique contracts; COP 42 trillion; 163,135 suppliers; 37 regional codes.

To avoid comparing incomplete calendar periods, all year-by-year descriptive analyses in the manuscript were closed at 31 December 2025. The platform continues to ingest newer records for operational monitoring, but post-2025 records were excluded from the formal analytical cohort reported here.

All primary monetary values are reported in COP. When approximate USD equivalents are provided in parentheses, they are secondary interpretive aids calculated using a round reference exchange rate of COP 4,000 per USD, close to late-2025 market levels. No analyses were performed in USD. Because a single round reference rate is applied across the entire 2020–2025 study period, the USD equivalents do not account for exchange-rate volatility and should be read as order-of-magnitude approximations only; the COP values are the analytical quantities. Detailed COP/USD reference conversions for the principal manuscript figures are provided in S1 Table.

**Fig 1:**
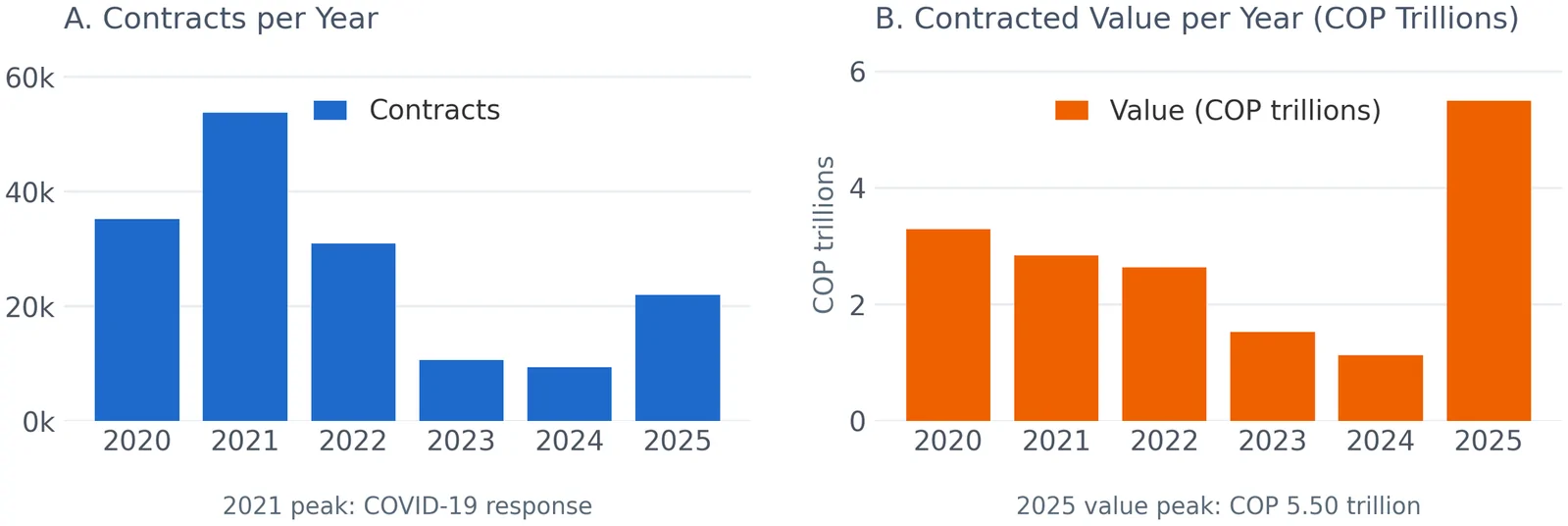
Annual evolution of public pharmaceutical procurement monitored in SECOP-II (closed cohort, 2020-2025): number of contracts and total contracted value.

**Fig 2:**
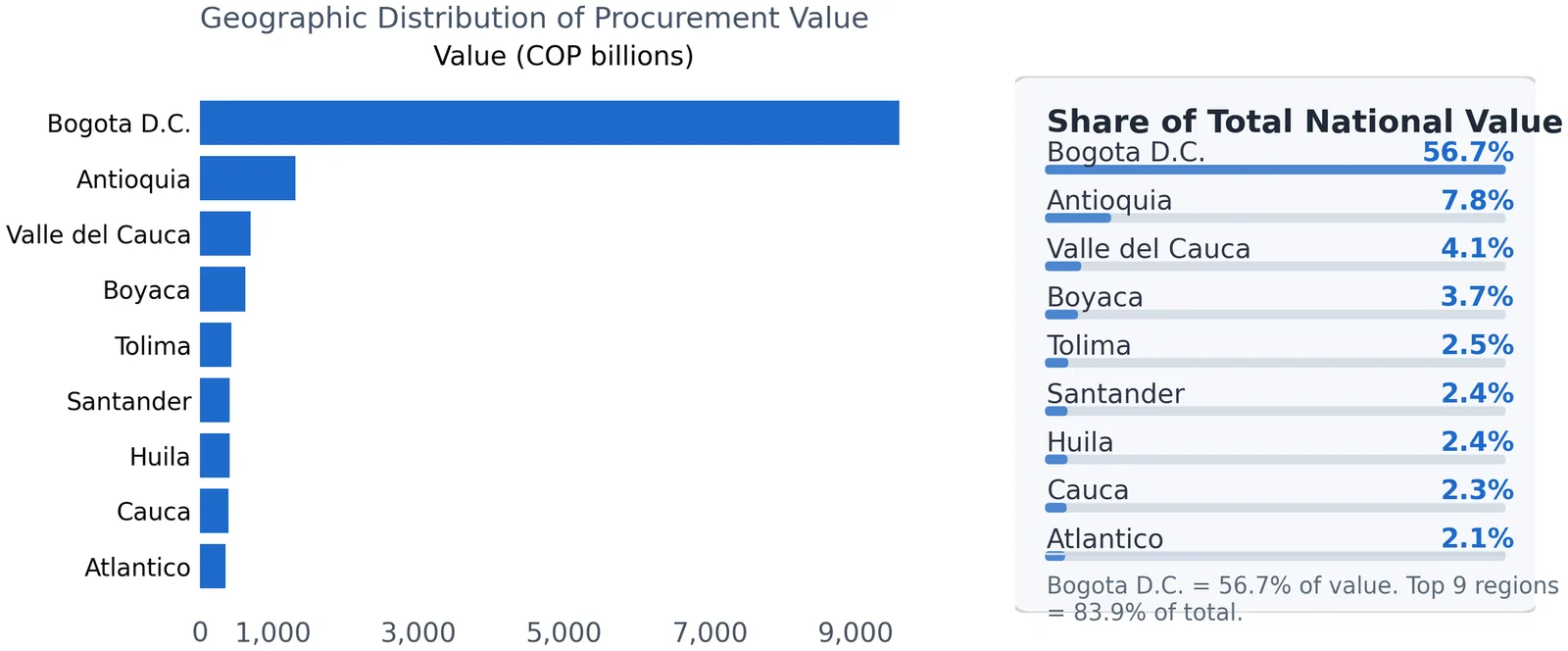
Geographic distribution of total value in public pharmaceutical procurement monitored in SECOP-II by department (closed cohort, 2020-2025).

**Fig 3:**
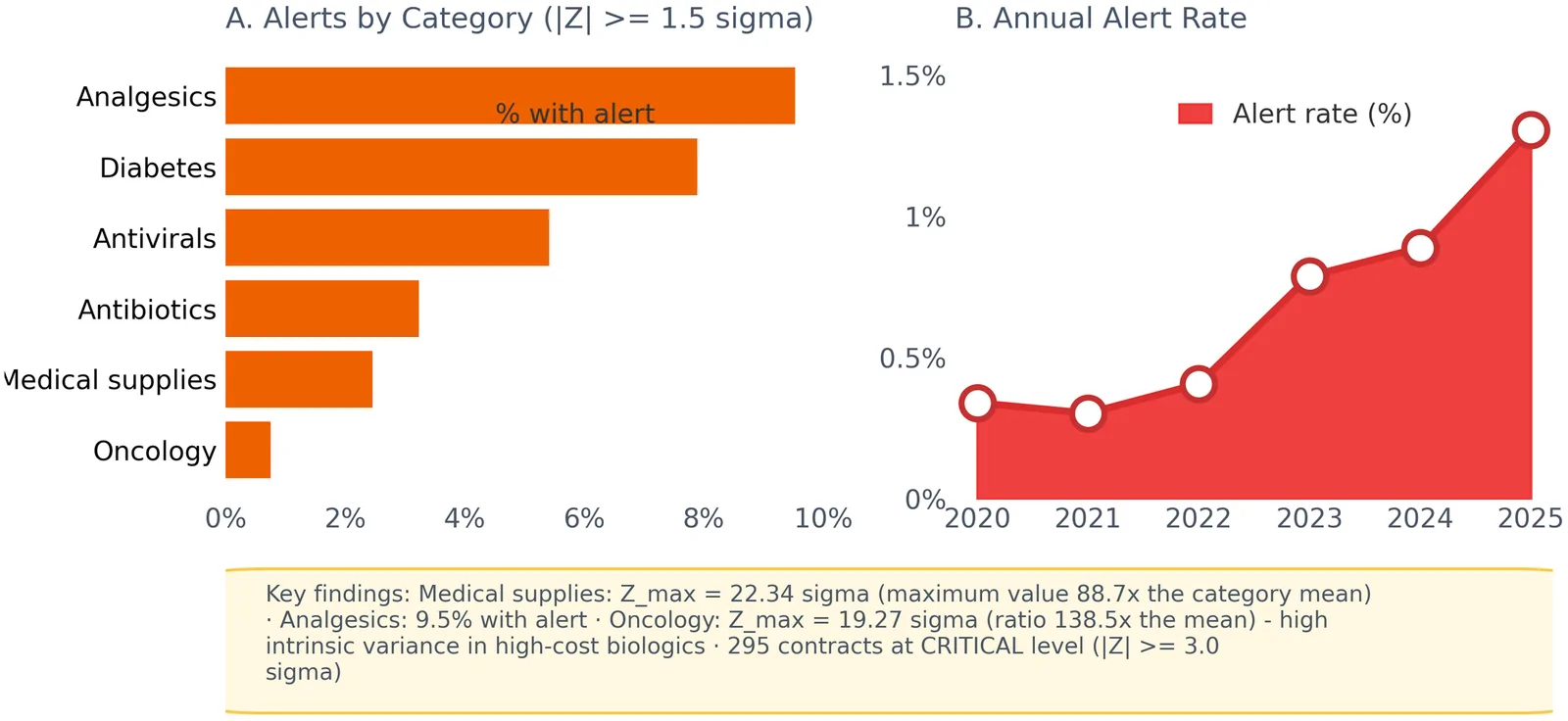
Distribution of statistical contract-value anomalies detected by the Z-score engine, by therapeutic category and year (2020-2025).

**Fig 4:**
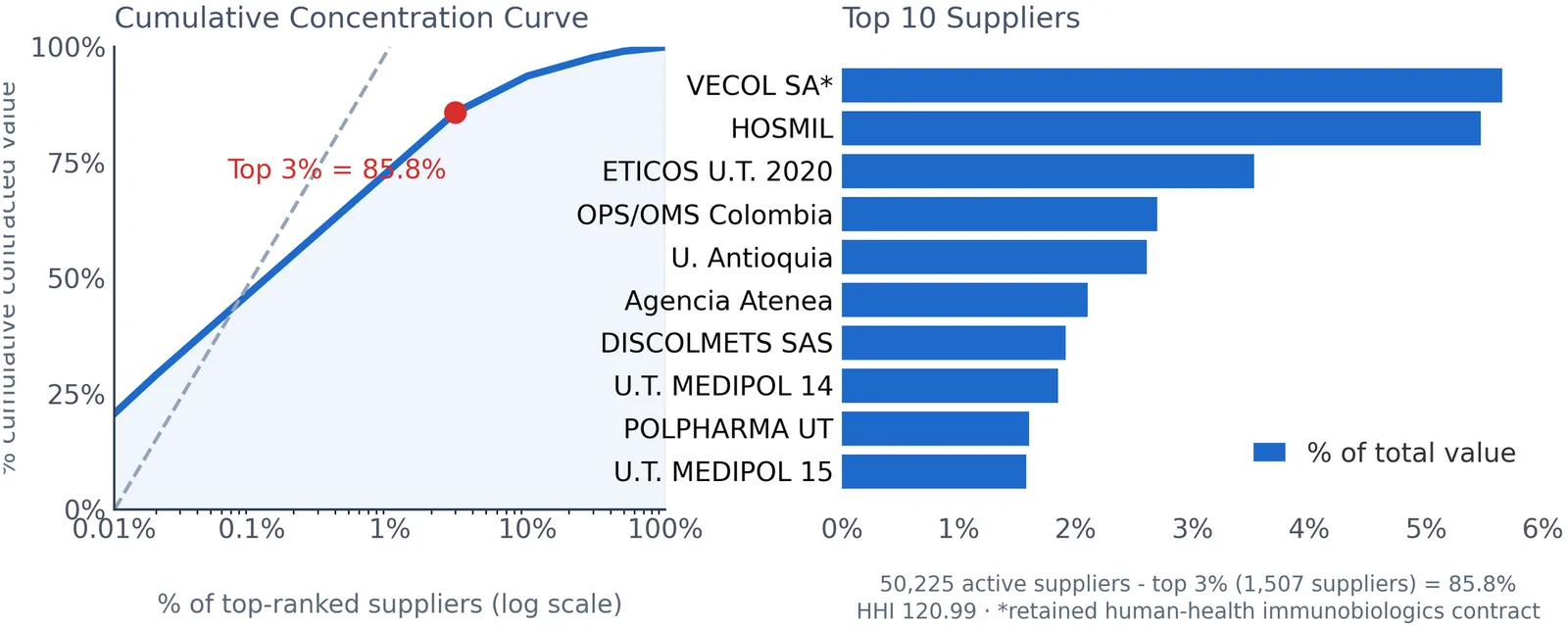
Market concentration in public pharmaceutical procurement monitored in SECOP-II (corrected closed cohort, 2020-2025): cumulative value concentration by top-ranked suppliers and top 10 supplier shares.

Source data are provided for Table 1 (S1 Data), Table 2 (S2 Data), Fig 1 (S3 Data), Fig 2 (S4 Data), Fig 3 category and yearly panels (S5 and S6 Data), and Fig 4 Lorenz and top-10 supplier panels (S7 and S8 Data); the column dictionary and source-file manifest are provided as S9 and S10 Data, and the SECOP-II filtering taxonomy, simplified database schema, and smart-contract protocol are provided as S1 Appendix.

**Table 1:** Summary of the public pharmaceutical procurement corpus and the closed analytical cohort used in this manuscript.

Variable	Value
Total SECOP-II contracts (API universe)	272,814
Pharmaceutical contracts in source closed cohort before contract-level review	162,271
Pharmaceutical contracts in corrected closed cohort	161,830
Total contracts in recovered local PostgreSQL source	956,157
Total contracted value (corrected pharmaceutical cohort)	COP 16.93 trillion (approx. USD 4.23 billion)
Total contracted value (all sectors recovered local source)	COP 294.7 trillion
Total contracted value (API universe)	COP 42.00 trillion
Mean value per positive-value pharmaceutical contract	COP 104.69 million
Unique normalized suppliers in corrected positive-value cohort	50,225
Regions covered	36-37
Period (closed pharmaceutical cohort)	2020–2025
Platform monitoring window	January 2015 onward
Processed INVIMA records	9,838 (all current)
SISMED reference prices	44,038 records; 1,759 unique ATC codes (2017–2019)
Indexed RAG documents	9,336

**Table 2:** Technical architecture of the BigLoI platform for surveillance of public pharmaceutical procurement.

Layer	Main technology	Function
Collection	Python; Socrata API	Incremental ingestion of SECOP-II, INVIMA, and SISMED
Storage	PostgreSQL; Pinecone; MongoDB	Relational, vector, and unstructured data storage
Processing	FastAPI; pandas	Cleaning, normalization, ABC classification, Z-score engine
Generative AI	Claude 3.5 Sonnet; GPT-4o	Hybrid semantic plus TF-IDF retrieval-augmented generation
Machine learning	scikit-learn	Demand prediction ($R^{\text{2}}>\text{0}.\text{8}\text{5}$), k-means (6 groups), PCA
Smart contracts (proof of concept)	Solidity; Chainlink CRE; Sepolia testnet	Simulated payment-state transitions across five digital states; invoice NFTs (secondary module)
Visualization	React/TypeScript	Public observatory with time series, maps, and alerts

**INVIMA (National Food and Drug Surveillance Institute).** A total of 9,838 records from the national sanitary registry were downloaded through the Socrata API. Variables included brand name, active ingredient, marketing authorization holder, sanitary registration number, ATC code, dosage form, route of administration, relevant dates, and registry status. All loaded records were current.

**SISMED (Medicine Price Information System, Ministry of Health).** We processed 44,038 reference-price records covering 1,759 unique ATC codes, aggregated by dosage form, distribution channel, and reference year (2017–2019). These data were used as contextual market references and as complementary input for interpreting atypical contract-value findings.

### 2.3 BigLoI technical architecture

The platform was implemented as a monorepo with a seven-layer architecture (Table 2).

**Fig 5:**
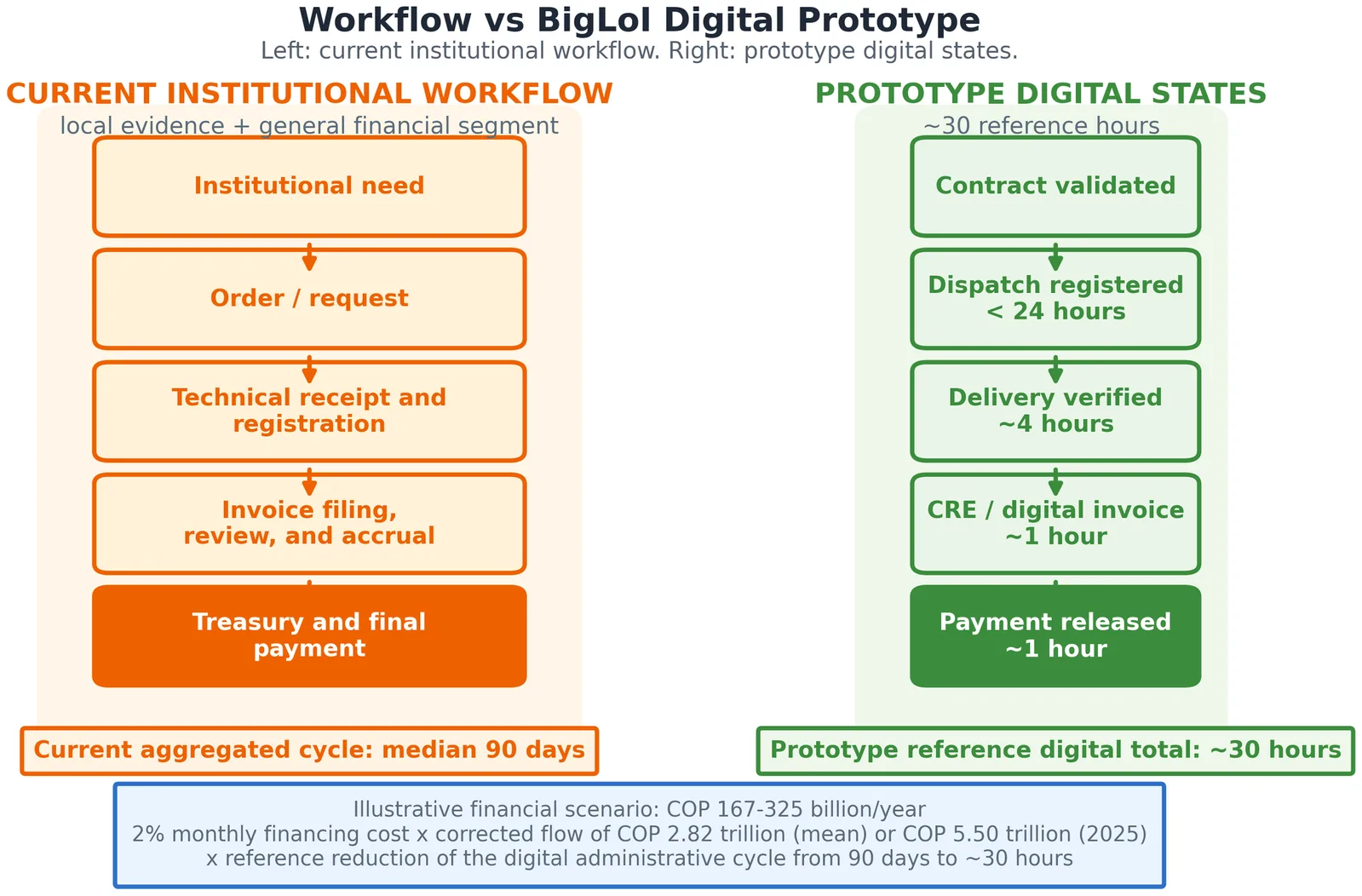
Conceptual scheme of the pharmaceutical payment workflow: current institutional flow versus digital states of the smart-contract prototype.

**Fig 6:**
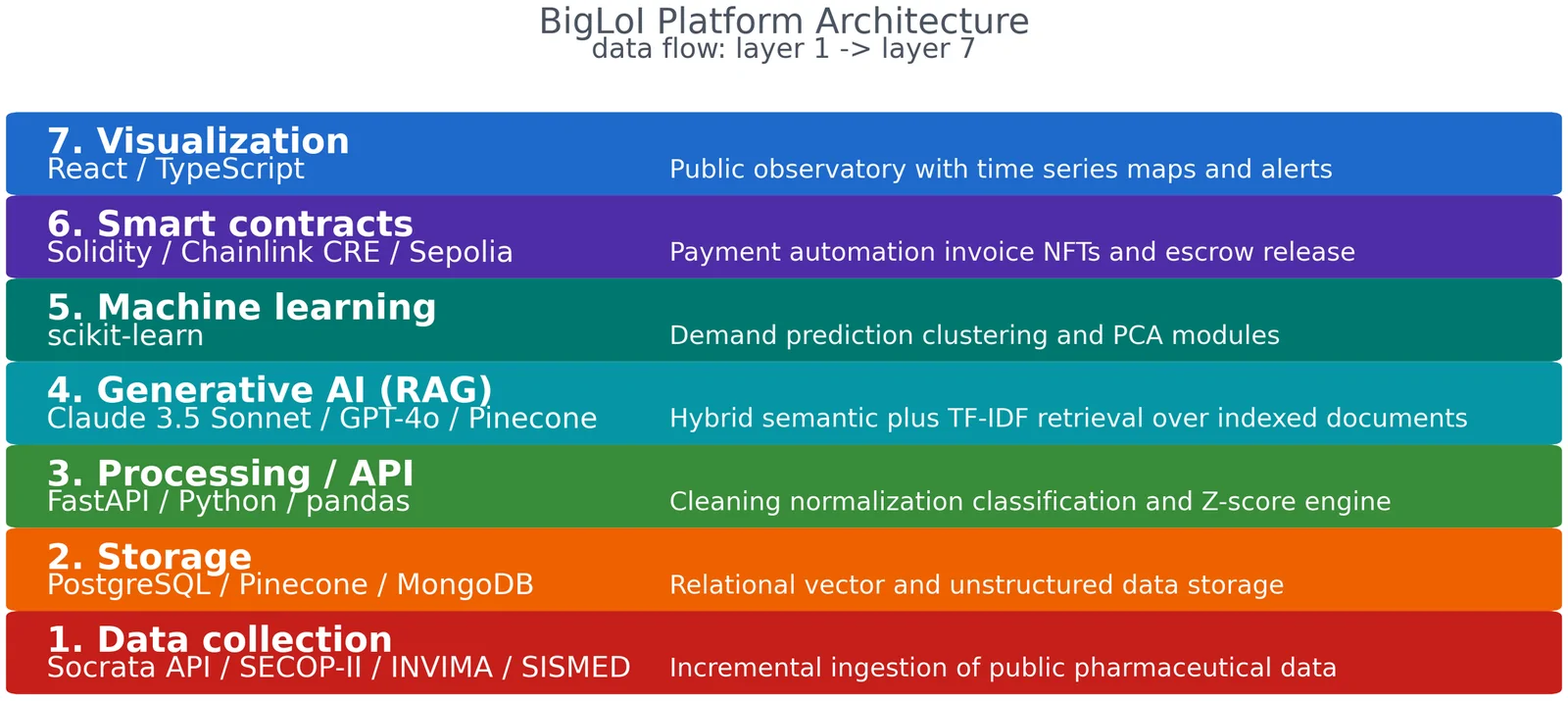
Simplified technical architecture of the BigLoI platform: seven layers for processing public pharmaceutical data.

### 2.4 Anomaly-detection engine based on Z-scores

For each pharmaceutical contract classified into a named therapeutic category, we calculated the standardized deviation of its total contract value relative to the distribution of observed values within the same category. Contracts labeled NO_ESPECIFICADO (unspecified therapeutic category) were excluded from the Z-score engine because they lack a stable within-category comparator. The analysis was further restricted to named categories with at least 10 contracts to avoid unstable estimates:


$$\begin{array}{c} Z_{i}=\frac{V_{i}-{\bar{V}}_{cat}}{\sigma_{cat}} \end{array}$$

where $Z_{i}$ is the anomaly score for contract i, $V_{i}$ is the contract’s total value, ${\bar{V}}_{cat}$ is the mean contract value within the same therapeutic category, and $\sigma_{cat}$ is the standard deviation of that category-specific distribution. This approach detects contracts with atypical total values relative to their category; by itself, it does not establish unit-price overpricing against a regulatory benchmark.

Alert levels were classified as follows:

**Table:** 

Level	Criterion	Interpretation
Critical	Absolute Z-score greater than or equal to 3.0 sigma	Extremely atypical contract value within category
High	Absolute Z-score from 2.0 to less than 3.0 sigma	Clearly atypical contract value
Moderate	Absolute Z-score from 1.5 to less than 2.0 sigma	Moderately atypical contract value
Normal	Absolute Z-score below 1.5 sigma	Within the expected range for category

### 2.5 Market-concentration analysis

To characterize the competitive structure of the pharmaceutical procurement market, we calculated descriptive concentration indicators and estimated the proportion of total contracted value accumulated by leading suppliers. Supplier concentration was calculated over positive-value contracts in the closed 2020–2025 pharmaceutical cohort. The Herfindahl-Hirschman Index (HHI) was calculated as the sum of squared supplier shares of total contracted value and is reported on the conventional 0–10,000 scale. Bipartite graphs of buyers and suppliers were constructed to identify recurrent contracting patterns.

### 2.6 Complementary technical-feasibility analysis of a payment workflow using smart contracts

As a clearly secondary module, we implemented a five-state simulator on the Sepolia testnet solely to verify that predefined digital states of a hypothetical payment workflow could be executed in sequence under controlled test conditions. It was not designed as an empirical time-and-motion study; comparison with current practice was made only at the level of temporal order of magnitude, using aggregated institutional references (90-day median; 60–180 day range) rather than a step-by-step operational equivalence between the full hospital workflow and the digital prototype states. The prototype reference scenario of approximately 30 hours should be read only as a test-condition benchmark for the simulator.

The illustrative projected savings were estimated as a scenario exercise, not as an empirical economic evaluation, by applying a conservative upper-bound monthly financing-cost assumption of 2% (approximately 26.8% effective annual) to annual contracted value under a hypothetical reduction in administrative delays. That rate was chosen as an order-of-magnitude working-capital parameter aligned with the upper range of legal interest ceilings derived from the Colombian *interés bancario corriente* certified by the Superintendencia Financiera de Colombia for ordinary/consumer credit, where maximum remuneratory or default interest equals 1.5 times the certified rate (usury ceiling) [11,12]. It is not an empirically measured average supplier financing rate in SECOP-II, nor a claim that pharmaceutical suppliers uniformly borrow at this cost; commercial or rediscount credit lines can be lower. Prolonged payment cycles in the Colombian pharmaceutical supply chain motivate treating delayed settlement as financially costly [13], but the 2% figure remains a scenario assumption only:


$$\begin{array}{c} Annual\;savings=V_{annual}\times r_{monthly}\times \frac{\Delta t_{days}}{\text{3}\text{0}} \end{array}$$

where $V_{annual}$ is either the corrected six-year mean annual flow (approximately COP 2.82 trillion) or the corrected 2025 flow (approximately COP 5.50 trillion), $r_{monthly}=\text{2}\%$, and Δt is the theoretical reduction in payment time, expressed in days, obtained when a 90-day cycle is shortened to approximately 30 hours (1.25 days), that is, approximately 88.75 days.

### 2.7 Statistical analysis

Descriptive statistics were used throughout. Log-normality of contract values was assessed with the Kolmogorov-Smirnov test. Market concentration was described using participation and accumulation indicators. The significance threshold was set at α=0.05.

## 3 Results

### 3.1 Characteristics of the data corpus

The characteristics of the corpus and the closed 2020–2025 analytical cohort are summarized in Table 1.

Pharmaceutical contract value followed a log-normal distribution (Kolmogorov-Smirnov D=0.04; p<0.001). The top 10% of contracts by value accounted for 78% of total contracted value.

**Annual evolution of indexed pharmaceutical contracts (closed cohort, 2020–2025):**

**Table:** 

Year	Contracts	Value (COP billions)
2020	35,242	3,294.5
2021	53,723	2,846.1
2022	30,927	2,639.6
2023	10,648	1,527.9
2024	9,335	1,124.0
2025	21,955	5,496.8

Fig 1 shows the annual evolution of the number of contracts and total contracted value across the closed 2020–2025 cohort. The peak in contract counts in 2021 is consistent with the COVID-19 response period, whereas the value peak in 2025 coincided with increased participation of high-cost oncology procurement. Post-2025 records remain available in the live platform but were excluded from the closed annual comparisons reported here.

The pronounced decline in indexed contracts in 2023–2024 (from 30,927 in 2022 to 10,648 and 9,335, respectively) is also present in an independent reconstruction of the public SECOP-II API, which recovered 10,006 and 8,818 candidate pharmaceutical contracts for those years before the contract-level correction. The decline is therefore present in the SECOP-II source under the inclusion taxonomy and is not an artifact of the local PostgreSQL snapshot. The available administrative data do not allow us to adjudicate whether it reflects procurement consolidation into fewer, higher-value contracts, changes in contract-object coding, or an actual reduction in pharmaceutical contracting volume; we therefore treat this pattern as a source-data and classification limitation requiring dedicated validation rather than assigning it a single causal explanation.

**Distribution by therapeutic category:**

**Table:** 

Category	Contracts	Value (COP billions)	Mean value (COP millions)
General pharmaceutical	81,589	9,855.7	120.8
Medical device	15,863	2,922.2	184.2
Unspecified (NO_ESPECIFICADO)	15,226	2,625.8	173.8
Vaccine	45,935	864.6	18.8
Oncology	400	421.2	1,052.9
Medical supply	2,232	190.0	85.1
Antibiotic	217	19.6	90.2
Diabetes	279	20.4	73.1
Analgesic	42	8.5	203.0
Antiviral	37	0.9	24.6

Unspecified contracts accounted for 9.4% of corrected-cohort contracts and 15.5% of total contracted value (COP 2.63 trillion). Oncology contracts showed the highest mean contract value among named categories (COP 1,052.9 million per contract). Contracts labeled NO_ESPECIFICADO are retained in descriptive cohort totals but are excluded from the Z-score engine (Section 2.4).

### 3.2 Geographic distribution

The leading regions by pharmaceutical procurement value were as follows:

**Table:** 

Region	Contracts	Value (COP billions)	Approx. value (USD millions)	% of total
Bogota D.C. (combined)	18,020	9,607.0	2,401.8	56.7%
Antioquia	47,964	1,312.2	328.1	7.8%
Valle del Cauca	5,843	696.5	174.1	4.1%
Boyaca	9,142	623.6	155.9	3.7%
Tolima	3,773	426.9	106.7	2.5%
Santander	4,278	403.8	101.0	2.4%
Huila	4,177	403.2	100.8	2.4%
Cauca	2,015	389.6	97.4	2.3%
Atlantico	5,075	348.8	87.2	2.1%

Bogota D.C. accounted for 56.7% of national contracted value, equivalent to approximately USD 2.40 billion at the reference exchange rate, followed by Antioquia (7.8%) and Valle del Cauca (4.1%). Fig 2 maps the geographic distribution of total contracted value by department.

**Methodological note.** Bogota D.C. appears under two regional codes in SECOP-II. Coverage of 36–37 regions exceeds the 32 official departments because SECOP-II includes distinct codings for districts and other territorial entities.

### 3.3 Contract-value anomalies detected by the Z-score engine

**Methodological note.** The implemented Z-score engine measures deviation of total contract value relative to the distribution within each therapeutic category. Direct comparison against SISMED unit prices would require structured line-item detail, which is not consistently available in SECOP-II.

Among 146,594 analyzed contracts in named therapeutic categories with at least 10 observations (after excluding 15,226 contracts labeled NO_ESPECIFICADO and 10 contracts in categories with fewer than 10 observations), the engine identified the following:

**Table:** 

Alert level	Criterion	Contracts	% of total
Critical	Absolute Z-score greater than or equal to 3.0 sigma	295	0.20%
High	Absolute Z-score from 2.0 to less than 3.0 sigma	213	0.15%
Moderate	Absolute Z-score from 1.5 to less than 2.0 sigma	156	0.11%
Total with alert	Absolute Z-score greater than or equal to 1.5 sigma	664	0.45%

These alerts flag contracts whose total value is atypical within their therapeutic category; they are intended for audit prioritization and do not, by themselves, constitute evidence of corruption, fraud, or unit-price overpricing.

**Results by therapeutic category:**

**Table:** 

Category	Contracts	% with alert	Zmax	Max/mean ratio
Analgesic	42	9.5%	4.22	9.3x
Diabetes	279	7.9%	6.61	15.5x
Antiviral	37	5.4%	4.83	14.2x
Antibiotic	217	3.2%	8.89	26.4x
Medical supply	2,232	2.5%	22.34	88.7x
Oncology	400	0.8%	19.27	138.5x

Among the six categories displayed in Fig 3, medical supplies showed the highest maximum Z-score, whereas analgesics showed the highest proportion of contracts with alerts.

**Temporal evolution of anomaly rates (2020–2025):**

**Table:** 

Year	Contracts analyzed	Contracts with alert	Rate
2020	35,238	120	0.34%
2021	53,718	163	0.30%
2022	30,926	126	0.41%
2023	10,648	84	0.79%
2024	9,335	83	0.89%
2025	6,729	88	1.31%

The statistical-alert rate among Z-score-eligible contracts increased from 0.30% in 2021 to 1.31% in 2025. Causal interpretation of this pattern requires additional analysis. The sharp reduction in the 2025 Z-score denominator (6,729 of 21,955 indexed contracts; 30.7%) is explained by categorical filtering rather than by incomplete data upload: all 15,226 NO_ESPECIFICADO contracts in the corrected closed cohort fall in 2025 and were excluded from the engine (21,955 − 6,729 = 15,226), whereas 2020–2024 retained near-complete coverage under the same rules (gaps of at most 10 contracts in those years, corresponding to named categories with fewer than 10 observations). Those unspecified 2025 contracts represent approximately COP 2.63 trillion—about 48% of 2025 contracted value and 15.5% of corrected-cohort value—so the 1.31% alert rate describes only the named-category slice analyzed by the engine and should not be read as an anomaly rate for all 2025 pharmaceutical procurement.

**Geographic distribution of anomalies.**

Departments with the highest rates of statistically flagged contracts were Bogota D.C. (3.80%), Norte de Santander (1.92%), Valle del Cauca (1.21%), and Tolima (1.00%). This distribution indicates heterogeneity in contract values, not causal evidence of corruption.

### 3.4 Market concentration

For value-share concentration metrics, only positive-value contracts with non-empty normalized supplier labels were included: 161,710 contracts and 50,225 suppliers (Table 1). Zero-value or blank-label rows were therefore outside the concentration denominator even when present in the corrected cohort. The top 10 suppliers concentrated 29.01% of total contracted value and the top 3% of suppliers (1,507 of 50,225) concentrated 85.83%. The leading normalized supplier label corresponded to one retained VECOL contract (5.65% of total value) whose object explicitly concerned a Ministry of Health–National Institute of Health agreement for research, development, and national production of human-health immunobiological products; veterinary VECOL contracts were excluded individually. The HHI was 120.99 on the 0–10,000 scale. The detailed distribution is shown in Fig 4.

This pattern indicates strong cumulative concentration among leading suppliers, while the HHI remains low under conventional market-concentration thresholds because the contracted value is distributed across a large long-tail supplier base. The bipartite graph identified 847 recurrent entity-supplier pairs with at least five consecutive contracts.

### 3.5 Smart-contract payment workflow: complementary technical proof of concept

Tests in the Sepolia-based BigLoI plus Chainlink CRE simulator verified that the prototype could execute predefined transitions between digital states. Fig 5 contrasts the current institutional payment flow with the digital states of the smart-contract prototype. Reported times are investigator-defined reference times used for functional validation of the prototype and do not represent observed measurements of the full real-world logistics and administrative process. SECOP-II was used as a contractual reference point rather than as an operational stage of hospital workflow, and no hospital payment system was instrumented or experimentally compared.

**Table:** 

Functional stage	Prototype time
Validated contract or enabled order to medicines dispatched	About 24 hours
Medicines dispatched to delivery verified	About 4 hours
Delivery verified to CRE registered	About 1 hour
CRE registered to payment released	About 1 hour
Full digital cycle of the prototype	About 30 hours

The current institutional cycle reported in the literature is 60–180 days (90-day median). This contrast is heuristic and order-of-magnitude only for the digital administrative component; it is not a direct equivalence to the full hospital process, nor evidence that the prototype would reproduce such reductions in practice.

**Illustrative financial-savings scenario.**

Average annual pharmaceutical procurement flow across the corrected closed 2020–2025 cohort was approximately COP 2.82 trillion (about USD 705 million at the reference exchange rate). Under a theoretical reduction from 90 days to approximately 30 hours and the conservative upper-bound 2% monthly financing-cost parameter described in Methods §2.6, the corresponding scenario output is approximately COP 167,000 million per year (USD 41.7 million). Using corrected 2025 flow, the illustrative output rises to approximately COP 325,000 million per year (USD 81.3 million). These values are scenario outputs only and should not be interpreted as observed savings, budget impact estimates, or implementation-ready business cases.

### 3.6 Complementary modules: machine learning and semantic retrieval

The complementary machine-learning and semantic-retrieval modules (layers 4 and 5 of the platform architecture in Fig 6) showed operational feasibility, but their outputs should be interpreted as exploratory within the scope of this paper.

## 4 Discussion

### 4.1 Scale and relevance of the documented problem

The corrected corpus of 161,830 pharmaceutical contracts from 2020 to 2025 represents, to our knowledge, the largest validated closed longitudinal series of public pharmaceutical procurement described for Colombia. The log-normal distribution of contract values, the concentration of 78% of total value in the top decile, and the HHI of 120.99 are consistent with procurement markets characterized by a small group of high-value suppliers and a large long-tail supplier base [14]. Because the HHI was computed for the national closed cohort as a whole, it may understate concentration that could exist within specific therapeutic groups (for example oncology, antibiotics, or antivirals); category-specific HHI values were not estimated in this study. Under conventional market-concentration thresholds, an HHI of 120.99 would be interpreted as unconcentrated at the national aggregate level; however, that reading can coexist with high cumulative value shares among leading suppliers (top 3% = 85.83%) when a large long-tail supplier base dilutes the index. Estimating category-specific HHI for oncology, antibiotics, antivirals, and related groups is left for future work and would be required before drawing subgroup competition inferences.

The increase in statistical-alert rates from 0.30% in 2021 to 1.31% in 2025 suggests increasing heterogeneity in contracted values during the study period. This pattern warrants dedicated investigation with adjustment for potential confounders such as pharmaceutical inflation and the introduction of new high-cost categories.

### 4.2 Z-scores as a surveillance instrument

The Z-score methodology offers advantages over manual auditing: it operates on large contract volumes, is reproducible, and does not require confidential information. At the same time, threshold-based Z-scores are imperfect screens. Without an adjudicated audit gold standard, sensitivity and specificity cannot be estimated. The exploratory |Z| ≥ 1.5 threshold favors prioritization recall and can increase false positives (statistically atypical but legitimate high-value contracts), while false negatives remain possible when categories are internally heterogeneous, when contracts fall in NO_ESPECIFICADO, or when category sample sizes are small. Future linkage with SISMED unit prices and item-level disaggregation would likely improve the interpretability of the detector [15,16]. The sustained increase in alert rates from 2021 to 2025 supports evaluation of this approach for routine audit prioritization workflows, not as a standalone determination of wrongdoing.

### 4.3 Smart contracts applied to pharmaceutical payment workflows: interpretation of a complementary proof of concept

The proof of concept supports only a narrow technical claim: conditional digital state transitions can be encoded and executed in a prototype payment workflow under testnet conditions. It does not demonstrate real-world reduction of payment times, lower transaction costs, or institutional deployability. The reported times are not measurements of the full payment process, which also depends on transport, packaging, storage, institutional validation, treasury procedures, contracting rules, and other bottlenecks not modeled here. A defensible operational evaluation would require prospective integration with public hospital information systems, real transaction traces, explicit counterfactual design, and independent technical and regulatory review. Large-scale implementation would additionally require public-treasury integration, legal validation, cybersecurity controls, and external smart-contract auditing.

### 4.4 Contribution to health data governance

Colombia has public data sources that can support pharmaceutical surveillance, but it lacks analytical infrastructure that reliably converts those data into actionable evidence. BigLoI illustrates that such infrastructure can be built using open technologies, reproducible workflows, and auditable queries over a contractual corpus.

### 4.5 Limitations

**SECOP-II data quality.** Buyer and supplier variables include null, generic, or inconsistent values in a meaningful share of contracts in local sample files, which constrains some network analyses. This reflects source-data quality issues in SECOP-II. The source closed cohort included 162,271 candidate contracts; contract-level review excluded 441 records with explicit veterinary, animal-health, or agricultural scope, yielding a corrected cohort of 161,830. The recovered local PostgreSQL source currently indexes 165,549 candidate pharmaceutical records including live 2026 monitoring rows.

**SISMED temporal mismatch and granularity.** The openly available SISMED source covers 2017–2019, whereas the main PostgreSQL procurement cohort analyzed here covers 2020–2025. Accordingly, SISMED was not used as a contemporaneous contract-by-contract comparator but as contextual market reference. In addition, the SECOP-II source does not provide a fully structured breakdown of line items and unit prices for all contracts, limiting direct validation of unit-price overpricing.

**Blockchain test-network scope.** As detailed in Section 4.3, the payment-workflow validation on Sepolia was a functional test of digital state transitions only: prototype times are referential, the savings scenario is not an economic evaluation, and the prototype was not tested against live institutional payment data. Mainnet implementation would require independent smart-contract auditing, regulatory validation, public-treasury integration, and cybersecurity review.

**Selection bias in pharmaceutical filtering.** Use of 19 inclusion keywords may generate false negatives and false positives. Contract-level review of 519 veterinary/agricultural candidates (441 excluded; 27 explicit human-health retained; 51 ambiguous retained) reduced a known contamination pathway, but the full keyword taxonomy has not been validated against an independently adjudicated reference set. A dedicated taxonomy-validation study is needed to quantify classification performance.

**Z-score false positives and false negatives.** As discussed in Section 4.2, statistical alerts prioritize atypical total-contract-value patterns within named categories and can yield both false positives and false negatives; they do not constitute evidence of corruption, fraud, or unit-price overpricing.

**Interpretive scope.** The observational design identifies statistical patterns but does not establish causal relationships or measure clinical impact. Interpreting flagged contracts as evidence of corruption or fraud would require additional adjudication methods and involvement of competent institutions.

### 4.6 Potential implications for public policy

These findings may inform three policy discussions—health-system reform, procurement transparency [17], and health-data governance—but they do not by themselves prescribe specific reforms. The principal contribution of the study is methodological and reproducible: an open infrastructure capable of transforming dispersed public data into auditable evidence. Concentration and recurrence indicators may support audit prioritization, and uneven SECOP-II data quality is a potential institutional concern for Colombia Compra Eficiente and related oversight bodies.

## 5 Conclusions

This study describes BigLoI, a national-scale computational pharmaceutical-surveillance platform that monitors SECOP-II contracts from 2015 onward and empirically analyzes a corrected closed cohort of 161,830 pharmaceutical contracts from 2020 to 2025. Three main findings emerge.

1. **Market concentration.** The top 3% of suppliers concentrated 85.83% of total contracted value, the top 10 suppliers concentrated 29.01%, and the HHI was 120.99 on the 0–10,000 scale.
2. **Atypical statistical patterns.** A total of 664 contracts triggered Z-score alerts with absolute Z-score greater than or equal to 1.5 sigma, antibiotics showed Zmax of 8.89, and the alert rate among Z-score-eligible contracts increased from 0.30% in 2021 to 1.31% in 2025. These alerts flag atypical total-value patterns for prioritization and do not constitute evidence of corruption or fraud; the 2025 rate applies only to named therapeutic categories after exclusion of NO_ESPECIFICADO.
3. **Reproducible infrastructure.** Integration of relational data storage, reproducible analytics, and a public observatory makes it possible to convert dispersed public sources into an auditable surveillance instrument for pharmaceutical procurement.

As a complementary result, the Sepolia prototype shows that conditional payment logic can be represented and executed in a controlled testnet environment under simulated conditions. Any inference about reduced payment delays or financial savings remains hypothetical and dependent on assumptions that were not empirically validated in this study. This line of work therefore remains exploratory and still requires operational, regulatory, technical, and logistical validation.

Overall, BigLoI shows that an open and reproducible surveillance infrastructure can generate descriptive findings on market concentration, statistical anomalies, and auditable evidence access from public administrative data on Colombian pharmaceutical procurement.

## Supporting information

S1 Table Approximate COP/USD equivalents for the principal monetary values reported in the manuscript. Conversions use a single round reference exchange rate of COP 4,000 per USD and are provided only as approximate interpretive aids; no analyses were performed in USD.
(DOCX)

S1 Data Source data for Table 1. (CSV)

S2 Data Source data for Table 2. (CSV)

S3 Data Source data for Fig 1. (CSV)

S4 Data Source data for Fig 2. (CSV)

S5 Data Source data for Fig 3 therapeutic-category panel. (CSV)

S6 Data Source data for Fig 3 yearly panel. (CSV)

S7 Data Source data for Fig 4 Lorenz curve. (CSV)

S8 Data Source data for Fig 4 top-10 supplier shares. (CSV)

S9 Data Column dictionary for the source-data files. (CSV)

S10 Data Source-file manifest for the uploaded supporting data files. (CSV)

S1 Appendix SECOP-II filtering taxonomy, simplified database schema, and smart-contract protocol. (DOCX)
