# Supplementary material for: Computational surveillance of Colombian public pharmaceutical procurement using public administrative data: A reproducible analysis of a closed 2020–2025 cohort
Source: PLoS One. 2026 Sep 11;21(9):e0350967. doi: 10.1371/journal.pone.0350967 (PMC13567785; doi:10.1371/journal.pone.0350967)
Supplement: Supplementary file 1 [file pone.0350967.s001.docx]

## Supplementary Table S1. Approximate COP/USD equivalences used as secondary interpretive references in the manuscript

Reference rule used throughout the manuscript: COP 4,000 = USD 1. These conversions are approximate interpretive aids only. All primary analyses, tables, and inferential statements were produced in COP rather than USD.

| Manuscript item | COP value | Approx. USD equivalent | Location in manuscript |
| --- | --- | --- | --- |
| Total contracted value of the corrected closed pharmaceutical cohort (2020-2025) | COP 16.93 trillion | USD 4.23 billion | Table 1 |
| Bogota D.C. combined procurement value | COP 9,607.0 billion | USD 2,401.8 million | Section 3.2 |
| Average annual pharmaceutical procurement flow across the corrected closed cohort | COP 2.82 trillion | USD 705 million | Section 3.5 |
| Illustrative annual savings scenario based on corrected average annual flow | COP 167,000 million | USD 41.7 million | Section 3.5 |
| Illustrative annual savings scenario based on corrected 2025 annual flow | COP 325,000 million | USD 81.3 million | Section 3.5 |

Interpretive note: these equivalences were included only to help non-Colombian readers gauge order of magnitude. They should not be interpreted as exchange-rate adjusted analyses, inflation-adjusted estimates, or purchasing-power comparisons.
