# Supplementary material for: Computational surveillance of Colombian public pharmaceutical procurement using public administrative data: A reproducible analysis of a closed 2020–2025 cohort
Source: PLoS One. 2026 Sep 11;21(9):e0350967. doi: 10.1371/journal.pone.0350967 (PMC13567785; doi:10.1371/journal.pone.0350967)
Supplement: Supplementary file 2 [file pone.0350967.s012.docx]

# S1 Appendix

## Appendix 1. SECOP-II pharmaceutical filtering taxonomy

Inclusion terms (19): medicamento, farmaceutico, farmacia, antibiotico, vacuna, insulina, quimioterapia, dispositivos medicos, insumo medico, suministro medico, biologico, antiviral, analgesico, anestesia, oncologico, hemodialisis, hormonal, anticoagulante, inmunosupresor.

Exclusion terms (5): obra civil, construccion, mantenimiento, tecnologia de informacion, mobiliario.

## Appendix 2. Simplified database schema

Main tables: observatorio_nacional.contratos_secop (956,157 total records in the recovered local snapshot; 165,549 candidate pharmaceutical records currently indexed; 162,271 in the source closed 2020-2025 cohort and 161,830 after contract-level veterinary exclusions); observatorio_nacional.sismed_precios_referencia (44,038 records; 1,759 unique ATC codes; years 2017-2019); observatorio_nacional.invima_medicamentos (9,838 current records); puntos_autorizados (101 hospitals); medicamentos (approximately 10,000 form records); sim_hospitales_100 (100 simulated hospitals).

## Appendix 3. Smart-contract protocol

Smart contracts: InvoiceRegistry.sol, InvoiceNFT.sol, and PaymentEscrow.sol; network: Ethereum Sepolia testnet; oracle layer: Chainlink CRE; invoice NFTs used for invoice traceability; escrow module used for conditional payment release.
