# Supplementary material for: Computational surveillance of Colombian public pharmaceutical procurement using public administrative data: A reproducible analysis of a closed 2020–2025 cohort
Source: PLoS One. 2026 Sep 11;21(9):e0350967. doi: 10.1371/journal.pone.0350967 (PMC13567785; doi:10.1371/journal.pone.0350967)
Supplement: Supplementary file 3 [file pone.0350967.s014.docx]

# Response to Reviewers

**Manuscript ID:** PONE-D-26-13579 **Title:** *Computational surveillance of Colombian public pharmaceutical procurement using public administrative data: a reproducible analysis of a closed 2020-2025 cohort* **Journal:** PLOS ONE **Corresponding author:** Andrés Soto (Independent researcher, Bogotá, Colombia; ORCID 0009-0004-8001-5372)

Dear Dr. Jose and Editors of PLOS ONE,

Thank you for the opportunity to submit a revised version of our manuscript. I am grateful for the careful editorial assessment and for the constructive comments from both reviewers, which have materially strengthened the manuscript.

I have revised the manuscript to address every journal requirement and reviewer comment. The revision (i) defines a single closed analytical cohort (complete calendar years 2020–2025) and reconciles all reported counts across five explicit data scopes; (ii) reframes the Z-score engine as an exploratory audit-prioritization instrument and reports threshold-stratified alert counts; (iii) acknowledges that sensitivity and specificity cannot be estimated without an adjudicated audit reference; (iv) adds the Herfindahl–Hirschman Index to the market-concentration analysis; (v) strengthens the treatment of the SISMED temporal mismatch and the absence of structured line-item pricing; (vi) removes causal/accusatory language; (vii) shortens and reframes the blockchain component as a secondary technical proof of concept; (viii) clarifies the currency convention and exchange-rate caveat; (ix) expands the ethics statement; and (x) adds in-text references to all figures and to Table 1 plus a Supporting Information section.

A point-by-point response follows. Reviewer comments are reproduced in summary form, followed by my response and the change made.

Sincerely, Andrés Soto

## Response to Journal Requirements

**JR-1 — Style and file naming.** The revised submission package follows PLOS ONE style and file-naming conventions: Manuscript.docx, Revised_Manuscript_with_Track_Changes.docx, Response_to_Reviewers.docx, Fig1.tif–Fig6.tif, and S1_Table.

**JR-2 — Ethics statement in Methods.** A full ethics statement has been added to Methods (§2.1): *“This study used only publicly accessible administrative procurement records published by Colombian open-data sources (SECOP-II via datos.gov.co, INVIMA, and SISMED). It did not involve human participants, patient-level or clinical data, identifiable private information, biological samples, recruitment, intervention, or any contact with individuals. Accordingly, the study does not constitute human-subjects research and did not require review or approval by an institutional review board or research ethics committee. Under Colombian Resolution 8430 of 1993, analysis of public, non-identifiable administrative records corresponds to research without risk and falls outside the scope of human-subjects ethics review. Informed consent was not applicable because the study did not involve human participants or identifiable personal data.”* The same statement was entered in the Editorial Manager Ethics Statement field.

**JR-3 — Title consistency.** The manuscript title and the Editorial Manager title now match exactly: *Computational surveillance of Colombian public pharmaceutical procurement using public administrative data: a reproducible analysis of a closed 2020-2025 cohort.*

**JR-4 — Abstract consistency.** The Editorial Manager abstract was updated to match the manuscript abstract exactly. Both describe the formal analytical cohort as the closed 2020–2025 cohort; post-2025 records are described only as live monitoring records and are not used in year-by-year comparisons.

**JR-5 — In-text figure references.** The revised text now refers explicitly to Figure 1 (§3.1), Figure 2 (§3.2), Figure 3 (§3.3), Figure 5 (§3.5), and Figure 6 (§2.3), in addition to the figure legends.

**JR-6 — In-text Table 1 reference.** Results §3.1 now states that the corpus characteristics and the closed 2020–2025 cohort are summarized in Table 1.

**JR-7 — Supporting Information captions.** A Supporting Information section was added at the end of the manuscript with the caption for **S1 Table** (approximate COP/USD equivalents), and the in-text citation was updated to the S1 Table format.

**JR-8 — Evaluation of suggested citations.** The reviewers did not provide a mandatory citation list. I reviewed the relevant methodological areas (market concentration/HHI, procurement-risk indicators, pharmaceutical market monitoring, and anomaly detection) and refined citations where they strengthened the Methods and Discussion.

## Response to Reviewer #1

**R1-1 — Cohort inconsistency (2020–March 2026 vs closed 2020–2025).** I agree. The reference to “2020 to March 2026” in the online abstract was a submission-metadata inconsistency, not a feature of the analysis. The formal analytical cohort comprises complete calendar years **2020–2025** (**162,271** pharmaceutical contracts); post-2025 records are retained only for live platform monitoring. *Change:* the manuscript and the Editorial Manager metadata now state the closed 2020–2025 cohort consistently, and a five-scope reconciliation (see R2-3) removes residual ambiguity.

**R1-2 — Justification of the |Z| ≥ 1.5σ threshold; sensitivity/specificity.** I agree. The revised Methods (§2.4) describe |Z| ≥ 1.5 as an **exploratory surveillance threshold** for prioritizing review in a high-volume administrative dataset, not a diagnostic threshold for wrongdoing; |Z| ≥ 2.0 and |Z| ≥ 3.0 are retained as higher-severity strata. Because no adjudicated gold-standard audit dataset exists for this corpus, sensitivity and specificity cannot be estimated directly; this is now stated explicitly. *Change:* Results §3.3 report the reconciled strata over **147,020** analyzed contracts — **685** alerts at |Z| ≥ 1.5, **528** at |Z| ≥ 2.0, and **303** at |Z| ≥ 3.0 — and the Limitations note the need for validation against adjudicated audit outcomes.

**R1-3 — 2023–2024 decline.** I agree this required clearer treatment. The revised Results §3.1 now ground the decline in evidence rather than assigning a single causal explanation. The pronounced drop in indexed contracts in 2023–2024 (from 30,991 in 2022 to 10,705 and 9,380) is reproduced in an independent reconstruction of the public SECOP-II API, which recovers 10,006 and 8,818 pharmaceutical contracts for those years (gaps of only 699 and 562 contracts relative to the manuscript counts). The decline is therefore present in the SECOP-II source under the inclusion taxonomy and is not an artifact of the local snapshot. Because the available administrative data do not allow us to adjudicate whether it reflects procurement consolidation into fewer, higher-value contracts, changes in contract-object coding, or an actual reduction in contracting volume, we treat it as a source-data and classification limitation requiring dedicated validation.

**R1-4 — Add the Herfindahl–Hirschman Index.** I agree. *Change:* Methods §2.5 specifies the HHI as the sum of squared supplier shares of total contracted value among positive-value contracts in the closed cohort, on the 0–10,000 scale; Results §3.4 report **HHI = 119.26**, alongside a top-10 share of **28.77%** and a top-3% share of **85.87%** (162,151 positive-value contracts; 50,355 suppliers).

**R1-5 — SISMED 2017–2019 limitation in Methods.** I agree. *Change:* Methods §2.2 now states explicitly that SISMED covers **2017–2019** and was used only as a contextual market reference, not as a contemporaneous contract-by-contract benchmark for the 2020–2025 cohort; the Limitations retain and expand this point.

**R1-6 — Keyword “artificial intelligence.”** I agree. *Change:* the keyword “artificial intelligence” was replaced with **“retrieval-augmented generation,”** which names the actual supporting AI component without overstating its analytical role.

**R1-7 — Figures must be high-quality with legible labels.** I agree; figures were treated as a resubmission priority. *Change:* Figures 1–6 were regenerated as high-quality raster files (TIFF) with all internal text in English and legible legends and annotations; figure source data remain in the reproducibility package.

**R1-8 — Single exchange rate / volatility warning.** I agree. *Change:* Methods §2.2 now states that COP is the analytical currency, that USD equivalents are approximate interpretive aids only, and that applying a single round reference rate (COP 4,000/USD) across 2020–2025 does not account for exchange-rate volatility; values are order-of-magnitude approximations. The conversion table is cited as S1 Table.

**R1-9 — Single independent author: external review or reproducibility.** I agree that independent verification matters. While the manuscript is single-authored, I strengthened the reproducibility package so that the analytical workflow can be independently inspected and replicated: source-data tables, figure and table source data, scripts, a column dictionary, a file manifest, and a Zenodo DOI, with the code repository openly available. *Change:* the Data and Code Availability section and relevant Methods text now emphasize auditability, openness, and reproducibility as the mechanisms supporting independent verification, and the single-author limitation is acknowledged explicitly.

## Response to Reviewer #2

**R2-1 — Z-score justification and sensitivity/specificity.** I agree; this is addressed jointly with R1-2. The Z-score engine is presented as an exploratory prioritization instrument, not a diagnostic classifier; threshold strata are reported (685 / 528 / 303), and the absence of sensitivity/specificity estimation is stated as a limitation, with validation against adjudicated audit outcomes proposed as future work. *Change:* Methods §2.4, Discussion §4.2, and Limitations were revised accordingly.

**R2-2 — Avoid overinterpretation; remain observational.** I agree. *Change:* Results, Discussion, Limitations, and Conclusions now use cautious observational language. Statistical alerts are described as atypical total-value patterns that may support audit prioritization, and the manuscript states explicitly that they cannot establish corruption, causal mechanisms, or unit-price overpricing without additional evidence.

**R2-3 — Discrepancies in contract counts.** I agree. *Change:* the manuscript now distinguishes five nested data scopes: (i) the SECOP-II monitored universe — 272,814 contracts (2015 onward); (ii) the recovered local PostgreSQL snapshot — 956,157 total records, of which 165,549 are pharmaceutical (including live post-2025 monitoring rows); (iii) the closed analytical cohort used here — **162,271** contracts (2020–2025); (iv) the value-share concentration subset — 162,151 positive-value contracts (50,355 normalized suppliers); and (v) the Z-score–analyzable subset — **147,020** contracts in categories with ≥10 observations. Earlier provisional figures (e.g., 162,921 contracts spanning 2020–March 2026) reflected a cohort that included partial 2026 records; closing the cohort to complete calendar years 2020–2025 yields 162,271. Methods §2.2 and the Table 1 footnote now state these scopes, and the manuscript, figures, source data, and reproducibility package were audited so that every count derives from a defined scope.

**R2-4 — Lack of structured line-item pricing.** I agree. *Change:* Methods §2.4 and the Limitations now state explicitly that the Z-score engine evaluates total contract values within therapeutic categories and cannot determine unit-price inflation or overpricing without structured item-level quantities, unit prices, dosage forms, and comparable specifications.

**R2-5 — Blockchain proof of concept is peripheral.** I agree. *Change:* the blockchain section (Methods §2.6, Results §3.5, Discussion §4.3) was shortened and reframed as a secondary technical-feasibility module tested on the Sepolia testnet only; the text states that it does not provide evidence of real-world payment-cycle reduction, realized savings, or implementation readiness.

**R2-6 — Conciseness.** I agree. *Change:* repetitive passages were condensed, particularly in the blockchain discussion, the interpretive-scope statements, the exchange-rate caveats, and the Limitations, while preserving the methodological clarifications requested by both reviewers.

## Summary of Major Revisions

1. Single closed 2020–2025 cohort (162,271 contracts) with a five-scope count reconciliation.
2. Z-score reframed as exploratory audit prioritization; threshold strata 685 / 528 / 303 over 147,020 analyzed contracts.
3. Explicit acknowledgment that sensitivity/specificity require an adjudicated reference set.
4. HHI added (119.26 on the 0–10,000 scale).
5. SISMED 2017–2019 mismatch and absence of line-item pricing emphasized in Methods and Limitations.
6. Causal/accusatory language removed throughout.
7. Blockchain shortened and framed as a secondary proof of concept.
8. Currency convention clarified with an exchange-rate volatility caveat.
9. Ethics statement expanded in Methods.
10. Figures 1–6 regenerated as high-quality English-language files.
11. Supporting Information section and in-text citations added.
12. Reproducibility package, Zenodo DOI, and open code emphasized for independent verification.
