# Supplementary material for: Computational surveillance of Colombian public pharmaceutical procurement using public administrative data: A reproducible analysis of a closed 2020–2025 cohort
Source: PLoS One. 2026 Sep 11;21(9):e0350967. doi: 10.1371/journal.pone.0350967 (PMC13567785; doi:10.1371/journal.pone.0350967)
Supplement: Supplementary file 4 [file pone.0350967.s015.docx]

# Response to Reviewers (Revision R2)

**Manuscript ID:** PONE-D-26-13579R1
**Title:** *Computational surveillance of Colombian public pharmaceutical procurement using public administrative data: a reproducible analysis of a closed 2020-2025 cohort*
**Journal:** PLOS ONE
**Corresponding author:** Andrés Soto (Independent researcher, Bogotá, Colombia; ORCID 0009-0004-8001-5372)

Dear Dr. Sun and Editors of PLOS ONE,

Thank you for the opportunity to revise the manuscript again and for the careful second-round assessment by both previous reviewers. I am grateful that the reviewers found the statistical analysis appropriate and the data fully available, and I have addressed every remaining clarification point below.

I verified the R1 package currently on file in Editorial Manager (PONE-D-26-13579_R1). That version matches the intermediate counts cited by the reviewers (for example **50,460** / **50,355** suppliers, **85.87%** top-3% concentration, **22,033** / **6,792** contracts for 2025, HHI **119.26**, and Z-score denominator **147,020**). The present revision retains every clarification requested below and additionally incorporates the final author-approved contract-level veterinary correction (dated 2026-07-11; previously disclosed to the editor): **161,830** contracts; **50,225** normalized suppliers; top-3% concentration **85.83%**; HHI **120.99**; and for 2025, **21,955** indexed contracts of which **6,729** enter the Z-score engine. All tables, figures, and reproducibility artifacts are aligned to these corrected figures.

A point-by-point response follows. Reviewer comments are summarized, followed by my response and the change made.

Sincerely,
Andrés Soto

## Response to Reviewer #1

**R1-1 — Source justifying the 2% per month financial rate (§2.6).**
I agree. *Change:* Methods §2.6 now frames the 2% monthly rate as a **conservative upper-bound scenario parameter** (approximately 26.8% effective annual), not as an empirically measured average sector rate. The quantitative anchor is the upper range of Colombian legal interest ceilings derived from the *interés bancario corriente* certified by the Superintendencia Financiera de Colombia, where maximum remuneratory or default interest equals 1.5 times that certified rate (usury ceiling) (refs 17–18). The text also notes that commercial or rediscount lines can be lower. As contextual motivation for treating payment delay as financially costly in the Colombian pharmaceutical supply chain—without claiming that AFIDRO measures a 2% monthly rate—we cite the AFIDRO/Sectorial receivables report on prolonged collection cycles (ref 19). Results §3.5 use the same “upper-bound scenario parameter” wording.

**R1-2 — Concentration 85.87% in text versus a conflicting Fig. 4 annotation (“Top 3% = 88.9%”).**
I agree that the text and Figure 4 must report the same concentration value. In the R1 Editorial Manager package, §3.4 reported **85.87%** while the Abstract/figure annotation used a rounded **85.9%**; the reviewer correctly flagged that the figure presentation was not acceptably synchronized with the text. *Change:* after the contract-level veterinary correction, text (§3.4, §5), source data, and Figure 4 are fully reconciled: the top 3% of suppliers concentrate **85.83%** of contracted value, and the figure annotation reads **“Top 3% = 85.8%”** (50,225 active suppliers; HHI 120.99).

**R1-3 — 50,460 versus 50,355 suppliers without explaining the transition.**
I agree that supplier scopes must be explicit. *Change:* Table 1 and §3.4 now use a single concentration denominator—**50,225** unique non-empty normalized supplier labels among **161,710** positive-value contracts—and §3.4 states that zero-value or blank-label rows are outside that denominator. The intermediate counts cited by the reviewer correspond to the pre-correction revision; they are superseded by the corrected figures above.

**R1-4 — Why only 6,792 of 22,033 indexed 2025 contracts entered the Z-score engine.**
I agree this required an explicit funnel explanation. The cause is **categorical filtering**, not incomplete data at upload: the contracts are present in the closed cohort but are ineligible for within-category Z-scores.

*Change:* Methods §2.4 now states that contracts labeled NO_ESPECIFICADO are excluded because they lack a stable within-category comparator, and that named categories with fewer than 10 contracts are also excluded. Results §3.1 now list NO_ESPECIFICADO in the therapeutic-category distribution (**15,226** contracts; **COP 2.63 trillion**; 15.5% of corrected-cohort value). Results §3.3 report the corrected 2025 funnel: **21,955** indexed → **6,729** analyzed (30.7%), because all **15,226** NO_ESPECIFICADO contracts fall in 2025 (21,955 − 6,729 = 15,226). Those unspecified contracts account for about **48% of 2025 contracted value**, so the 1.31% alert rate describes only the named-category slice and must not be read as an anomaly rate for all 2025 pharmaceutical procurement. Coverage for 2020–2024 remains near-complete under the same rules (aggregate gap of 10 contracts in categories with fewer than 10 observations).

**R1-5 — Limitation of national HHI (§4.1).**
I agree. *Change:* Section 4.1 now notes that the national HHI may understate concentration within specific therapeutic groups (for example oncology, antibiotics, or antivirals) and that category-specific HHI values were not estimated. It further clarifies that, under conventional thresholds, HHI = 120.99 would read as unconcentrated at the national aggregate level, yet that interpretation can coexist with the observed top-3% cumulative share of 85.83% when a large long-tail supplier base dilutes the index, and that subgroup HHI estimation is left for future work.

## Response to Reviewer #2

**R2-1 — Discuss Z-score limitations, including false positives and false negatives, in greater detail.**
I agree. *Change:* Section 4.2 and the Limitations now expand this point: without an adjudicated audit gold standard, sensitivity and specificity cannot be estimated; the exploratory |Z| ≥ 1.5 threshold favors prioritization recall and can increase false positives; false negatives remain possible under category heterogeneity, NO_ESPECIFICADO exclusion, or small category sizes.

**R2-2 — Keep clear that statistical alerts are not evidence of corruption or fraud.**
I agree. *Change:* this distinction is reinforced in Results §3.3, Discussion/Limitations, Abstract Conclusions, and Conclusions §5, which state explicitly that alerts are exploratory prioritization signals and do not constitute evidence of corruption or fraud.

**R2-3 — Present the smart-contract component cautiously as a simulated proof of concept.**
I agree. *Change:* the manuscript continues to frame the Sepolia module as a secondary technical-feasibility proof of concept under simulated conditions only. Abstract Results no longer foreground illustrative COP savings figures; they state only that predefined digital state transitions were executed under testnet conditions and that the module provides no evidence of real-world payment-cycle reduction, realized savings, or institutional deployability. Abstract Conclusions and §5 retain the same caution. Detailed scenario arithmetic, where retained, remains confined to Methods §2.6 / Results §3.5 as an explicitly non-empirical illustration.

**R2-4 — Additional information on validation of the keyword-based pharmaceutical filter.**
I agree. *Change:* Methods §2.2 now describes the 19-keyword screen as high-recall rather than gold-standard; explains false-positive/false-negative pathways; and reports the contract-level quality-control review of 519 candidates (441 excluded; 27 human-health retained; 51 ambiguous retained), while stating that no independently adjudicated external reference set was available for full taxonomy sensitivity/specificity. The Limitations retain the call for dedicated taxonomy validation.

**R2-5 — Frame policy-related statements as potential implications rather than direct conclusions.**
I agree. *Change:* Section 4.6 is retitled “Potential implications for public policy” and uses cautious language (“may inform,” “potential institutional concern”). Conclusions and the Abstract Conclusions retain descriptive findings and avoid prescribing reforms.

## Summary of Major Revisions (R2)

1. Justified the 2%/month financing-cost assumption as an upper-bound scenario parameter anchored to Colombian Superintendencia Financiera / usury ceilings (refs 17–18), with AFIDRO receivables context (ref 19).
2. Reconciled concentration reporting and Figure 4 at **85.83% / 85.8%** with **50,225** suppliers.
3. Added an explicit supplier-scope bridge for the concentration denominator (§3.4; Table 1).
4. Documented the 2025 Z-score funnel (NO_ESPECIFICADO exclusion; 21,955 → 6,729), including COP 2.63 trillion / ~48% of 2025 value and a cautious reading of the 1.31% alert rate.
5. Strengthened the national-HHI limitation regarding therapeutic subgroups and the coexistence of low aggregate HHI with high top-3% cumulative share (§4.1).
6. Expanded Z-score false-positive/false-negative discussion and reinforced that alerts ≠ corruption/fraud.
7. Strengthened keyword-filter validation disclosure (§2.2) and reframed policy text as potential implications.
