# Supplementary material for: Computational surveillance of Colombian public pharmaceutical procurement using public administrative data: A reproducible analysis of a closed 2020–2025 cohort
Source: PLoS One. 2026 Sep 11;21(9):e0350967. doi: 10.1371/journal.pone.0350967 (PMC13567785; doi:10.1371/journal.pone.0350967)
Supplement: Peer review history

# Peer review: Decision Letter 0

**DOI:** 10.1371/journal.pone.0350967.r001

**Authors:** Sarah Jose

2 Jun 2026

Dear Dr. Soto,

Please submit your revised manuscript by Jul 17 2026 11:59PM. If you will need more time than this to complete your revisions, please reply to this message or contact the journal office at plosone@plos.org. When you're ready to submit your revision, log on to https://www.editorialmanager.com/pone/ and select the 'Submissions Needing Revision' folder to locate your manuscript file.

- A letter that responds to each point raised by the academic editor and reviewer(s). You should upload this letter as a separate file labeled 'Response to Reviewers'.
- A marked-up copy of your manuscript that highlights changes made to the original version. You should upload this as a separate file labeled 'Revised Manuscript with Track Changes'.
- An unmarked version of your revised paper without tracked changes. You should upload this as a separate file labeled 'Manuscript'.

If you would like to make changes to your financial disclosure, please include your updated statement in your cover letter. Guidelines for resubmitting your figure files are available below the reviewer comments at the end of this letter.

If applicable, we recommend that you deposit your laboratory protocols in protocols.io to enhance the reproducibility of your results. Protocols.io assigns your protocol its own identifier (DOI) so that it can be cited independently in the future. For instructions see: https://journals.plos.org/plosone/s/submission-guidelines#loc-laboratory-protocols. Additionally, PLOS ONE offers an option for publishing peer-reviewed Lab Protocol articles, which describe protocols hosted on protocols.io. Read more information on sharing protocols at https://plos.org/protocols?utm_medium=editorial-email&utm_source=authorletters&utm_campaign=protocols.

As the corresponding author, your ORCID iD is verified in the submission system and will appear in the published article. PLOS supports the use of ORCID, and we encourage all coauthors to register for an ORCID iD and use it as well. Please encourage your coauthors to verify their ORCID iD within the submission system before final acceptance, as unverified ORCID iDs will not appear in the published article. *Only*  the individual author can complete the verification step; PLOS staff *cannot*  verify ORCID iDs on behalf of authors.

We look forward to receiving your revised manuscript.

Kind regards,

Sarah Jose, Ph.D.

Staff Editor

PLOS One

Journal Requirements:

When submitting your revision, we need you to address these additional requirements.

1. Please ensure that your manuscript meets PLOS ONE's style requirements, including those for file naming. The PLOS ONE style templates can be found at

https://journals.plos.org/plosone/s/file?id=wjVg/PLOSOne_formatting_sample_main_body.pdf and https://journals.plos.org/plosone/s/file?id=ba62/PLOSOne_formatting_sample_title_authors_affiliations.pdf

2. Please include your full ethics statement in the ‘Methods’ section of your manuscript file. In your statement, please include the full name of the IRB or ethics committee who approved or waived your study, as well as whether or not you obtained informed written or verbal consent. If consent was waived for your study, please include this information in your statement as well.

3. Please amend either the title on the online submission form (via Edit Submission) or the title in the manuscript so that they are identical.

4. Please amend either the abstract on the online submission form (via Edit Submission) or the abstract in the manuscript so that they are identical.

5. Please ensure that you refer to Figure 1, 2, 3, 5, 6, in your text as, if accepted, production will need this reference to link the reader to the figure.

6. We note you have included a table to which you do not refer in the text of your manuscript. Please ensure that you refer to Table 1 in your text; if accepted, production will need this reference to link the reader to the Table.

7. Please include captions for your Supporting Information files at the end of your manuscript, and update any in-text citations to match accordingly. Please see our Supporting Information guidelines for more information: http://journals.plos.org/plosone/s/supporting-information.

8. If the reviewer comments include a recommendation to cite specific previously published works, please review and evaluate these publications to determine whether they are relevant and should be cited. There is no requirement to cite these works unless the editor has indicated otherwise.

[Note: HTML markup is below. Please do not edit.]

Reviewers' comments:

Reviewer's Responses to Questions

**Comments to the Author**

1. Is the manuscript technically sound, and do the data support the conclusions?

Reviewer #1: Partly

Reviewer #2: Partly

**********

2. Has the statistical analysis been performed appropriately and rigorously?

Reviewer #1: Yes

Reviewer #2: Yes

**********

3. Have the authors made all data underlying the findings in their manuscript fully available?

The PLOS Data policy

Reviewer #1: No

Reviewer #2: Yes

**********

4. Is the manuscript presented in an intelligible fashion and written in standard English?

Reviewer #1: Yes

Reviewer #2: Yes

**********

Reviewer #1: - The abstract in Editorial Manager refers to a “primary analytical cohort from 2020 to March 2026,” whereas the main text of the manuscript consistently uses the term “closed cohort for 2020–2025,” which clearly excludes incomplete records for 2026.

- The choice of an alert threshold at |Z| ≥ 1.5σ is not justified by either a reference to the literature or a sensitivity-specificity analysis.

- The data in Table 1 (Section 3.1) show a sharp decline in the number of contracts in 2023 and 2024, followed by a partial recovery in 2025. The author cites the peak of the COVID-19 pandemic in 2021 and an increase in oncology procurement in 2025, but the nearly fivefold drop in 2023–2024 remains unexplained.

- The analysis of market concentration is limited to descriptive indicators (top 3%, top 10 suppliers…). To align with standards in economic and competition literature, it is recommended to supplement the analysis with, for example, the Herfindahl–Hirschman Index (HHI).

- The only publicly available SISMED dataset covers the years 2017–2019, whereas the analytical cohort spans 2020–2025. The authors note that SISMED was used merely as a “contextual market benchmark,” but this limitation should also be included in the “Methods” section.

- List of keywords includes the term “artificial intelligence,” but the manuscript primarily employs statistical methods, and the use of AI components (Claude 3.5 Sonnet, GPT-4o) serves as a supporting tool for the platform rather than the primary analytical method. It is recommended to replace “artificial intelligence” with a more precise term.

- All illustrations must be replaced with vector or high-quality raster files that include legible captions and legends. The resolution must meet PLOS One’s requirements.

- The author uses a single exchange rate of 4,000 USD for the entire analysis period—an explicit warning about exchange rate volatility must be added in the relevant methodological note (not only in Supplementary Table S1).

- The article is submitted by a single author who is an independent researcher. Given the scale and complexity of the work performed, it is recommended to either indicate external peer review of the methodology (e.g., by academic colleagues) or emphasize in the text how reproducibility and code openness compensate for the lack of co-author verification.

Reviewer #2: The manuscript provides a relevant analysis of Colombian public pharmaceutical procurement using open administrative data within a reproducible framework. It addresses key issues in public health governance and procurement transparency. The study is well-organised and demonstrates significant technical effort in integrating SECOP-II, INVIMA, and SISMED data.

Strengths include a large-scale longitudinal dataset, a focus on reproducibility, public data integration, and an honest acknowledgement of limitations. The conclusions regarding market concentration and unusual procurement patterns are well-supported by the data. The authors wisely avoid equating statistical anomalies with corruption, maintaining the exploratory nature of their analyses.

However, there are areas for improvement:

1. Statistical Methodology: The reliance on Z-score analysis for anomaly detection needs more methodological justification for the chosen thresholds and a discussion on sensitivity and specificity. Future work could benefit from advanced detection methods.

2. Interpretation of Anomalies: The manuscript should avoid overinterpreting findings and remain observational without causal implications.

3. Consistency and Reproducibility: Minor discrepancies in reported contract counts need clarification for better reproducibility.

4. Data Limitations: The lack of structured line-item pricing data limits the assessment of unit-price inflation and should be emphasised more clearly.

5. Blockchain Proof-of-Concept: While technically interesting, this section is peripheral to the main objectives and could be shortened.

6. Language and Readability: The manuscript is generally clear, but some repetitive sections could benefit from editing for conciseness.

There are no obvious ethical concerns regarding plagiarism or research ethics, and the study transparently uses publicly available datasets.

**********

what does this mean?). If published, this will include your full peer review and any attached files.

If you choose “no”, your identity will remain anonymous but your review may still be made public.

**Do you want your identity to be public for this peer review?**  For information about this choice, including consent withdrawal, please see our Privacy Policy

Reviewer #1: No

Reviewer #2: No

**********

[NOTE: If reviewer comments were submitted as an attachment file, they will be attached to this email and accessible via the submission site. Please log into your account, locate the manuscript record, and check for the action link "View Attachments". If this link does not appear, there are no attachment files.]

To ensure your figures meet our technical requirements, please review our figure guidelines: https://journals.plos.org/plosone/s/figures

You may also use PLOS’s free figure tool, NAAS, to help you prepare publication quality figures: https://journals.plos.org/plosone/s/figures#loc-tools-for-figure-preparation.

NAAS will assess whether your figures meet our technical requirements by comparing each figure against our figure specifications.

# Author comment: Author response to Decision Letter 1

**DOI:** 10.1371/journal.pone.0350967.r002

11 Jun 2026

Response to Reviewers

Manuscript ID: PONE-D-26-13579 Title: Computational surveillance of Colombian public pharmaceutical procurement using public administrative data: a reproducible analysis of a closed 2020-2025 cohort Journal: PLOS ONE Corresponding author: Andrés Soto (Independent researcher, Bogotá, Colombia; ORCID 0009-0004-8001-5372)

------------------------------------------------------------------------

Dear Dr. Jose and Editors of PLOS ONE,

Thank you for the opportunity to submit a revised version of our manuscript. I am grateful for the careful editorial assessment and for the constructive comments from both reviewers, which have materially strengthened the manuscript.

I have revised the manuscript to address every journal requirement and reviewer comment. The revision (i) defines a single closed analytical cohort (complete calendar years 2020–2025) and reconciles all reported counts across five explicit data scopes; (ii) reframes the Z-score engine as an exploratory audit-prioritization instrument and reports threshold-stratified alert counts; (iii) acknowledges that sensitivity and specificity cannot be estimated without an adjudicated audit reference; (iv) adds the Herfindahl–Hirschman Index to the market-concentration analysis; (v) strengthens the treatment of the SISMED temporal mismatch and the absence of structured line-item pricing; (vi) removes causal/accusatory language; (vii) shortens and reframes the blockchain component as a secondary technical proof of concept; (viii) clarifies the currency convention and exchange-rate caveat; (ix) expands the ethics statement; and (x) adds in-text references to all figures and to Table 1 plus a Supporting Information section.

A point-by-point response follows. Reviewer comments are reproduced in summary form, followed by my response and the change made.

Sincerely, Andrés Soto

------------------------------------------------------------------------

Response to Journal Requirements

JR-1 — Style and file naming. The revised submission package follows PLOS ONE style and file-naming conventions: Manuscript.docx, Revised_Manuscript_with_Track_Changes.docx, Response_to_Reviewers.docx, Fig1.tif–Fig6.tif, and S1_Table.

JR-2 — Ethics statement in Methods. A full ethics statement has been added to Methods (§2.1): “This study used only publicly accessible administrative procurement records published by Colombian open-data sources (SECOP-II via datos.gov.co, INVIMA, and SISMED). It did not involve human participants, patient-level or clinical data, identifiable private information, biological samples, recruitment, intervention, or any contact with individuals. Accordingly, the study does not constitute human-subjects research and did not require review or approval by an institutional review board or research ethics committee. Under Colombian Resolution 8430 of 1993, analysis of public, non-identifiable administrative records corresponds to research without risk and falls outside the scope of human-subjects ethics review. Informed consent was not applicable because the study did not involve human participants or identifiable personal data.” The same statement was entered in the Editorial Manager Ethics Statement field.

JR-3 — Title consistency. The manuscript title and the Editorial Manager title now match exactly: Computational surveillance of Colombian public pharmaceutical procurement using public administrative data: a reproducible analysis of a closed 2020-2025 cohort.

JR-4 — Abstract consistency. The Editorial Manager abstract was updated to match the manuscript abstract exactly. Both describe the formal analytical cohort as the closed 2020–2025 cohort; post-2025 records are described only as live monitoring records and are not used in year-by-year comparisons.

JR-5 — In-text figure references. The revised text now refers explicitly to Figure 1 (§3.1), Figure 2 (§3.2), Figure 3 (§3.3), Figure 5 (§3.5), and Figure 6 (§2.3), in addition to the figure legends.

JR-6 — In-text Table 1 reference. Results §3.1 now states that the corpus characteristics and the closed 2020–2025 cohort are summarized in Table 1.

JR-7 — Supporting Information captions. A Supporting Information section was added at the end of the manuscript with the caption for S1 Table (approximate COP/USD equivalents), and the in-text citation was updated to the S1 Table format.

JR-8 — Evaluation of suggested citations. The reviewers did not provide a mandatory citation list. I reviewed the relevant methodological areas (market concentration/HHI, procurement-risk indicators, pharmaceutical market monitoring, and anomaly detection) and refined citations where they strengthened the Methods and Discussion.

------------------------------------------------------------------------

Response to Reviewer #1

R1-1 — Cohort inconsistency (2020–March 2026 vs closed 2020–2025). I agree. The reference to “2020 to March 2026” in the online abstract was a submission-metadata inconsistency, not a feature of the analysis. The formal analytical cohort comprises complete calendar years 2020–2025 (162,271 pharmaceutical contracts); post-2025 records are retained only for live platform monitoring. Change: the manuscript and the Editorial Manager metadata now state the closed 2020–2025 cohort consistently, and a five-scope reconciliation (see R2-3) removes residual ambiguity.

R1-2 — Justification of the |Z| ≥ 1.5σ threshold; sensitivity/specificity. I agree. The revised Methods (§2.4) describe |Z| ≥ 1.5 as an exploratory surveillance threshold for prioritizing review in a high-volume administrative dataset, not a diagnostic threshold for wrongdoing; |Z| ≥ 2.0 and |Z| ≥ 3.0 are retained as higher-severity strata. Because no adjudicated gold-standard audit dataset exists for this corpus, sensitivity and specificity cannot be estimated directly; this is now stated explicitly. Change: Results §3.3 report the reconciled strata over 147,020 analyzed contracts — 685 alerts at |Z| ≥ 1.5, 528 at |Z| ≥ 2.0, and 303 at |Z| ≥ 3.0 — and the Limitations note the need for validation against adjudicated audit outcomes.

R1-3 — 2023–2024 decline. I agree this required clearer treatment. The revised Results §3.1 now ground the decline in evidence rather than assigning a single causal explanation. The pronounced drop in indexed contracts in 2023–2024 (from 30,991 in 2022 to 10,705 and 9,380) is reproduced in an independent reconstruction of the public SECOP-II API, which recovers 10,006 and 8,818 pharmaceutical contracts for those years (gaps of only 699 and 562 contracts relative to the manuscript counts). The decline is therefore present in the SECOP-II source under the inclusion taxonomy and is not an artifact of the local snapshot. Because the available administrative data do not allow us to adjudicate whether it reflects procurement consolidation into fewer, higher-value contracts, changes in contract-object coding, or an actual reduction in contracting volume, we treat it as a source-data and classification limitation requiring dedicated validation.

R1-4 — Add the Herfindahl–Hirschman Index. I agree. Change: Methods §2.5 specifies the HHI as the sum of squared supplier shares of total contracted value among positive-value contracts in the closed cohort, on the 0–10,000 scale; Results §3.4 report HHI = 119.26, alongside a top-10 share of 28.77% and a top-3% share of 85.87% (162,151 positive-value contracts; 50,355 suppliers).

R1-5 — SISMED 2017–2019 limitation in Methods. I agree. Change: Methods §2.2 now states explicitly that SISMED covers 2017–2019 and was used only as a contextual market reference, not as a contemporaneous contract-by-contract benchmark for the 2020–2025 cohort; the Limitations retain and expand this point.

R1-6 — Keyword “artificial intelligence.” I agree. Change: the keyword “artificial intelligence” was replaced with “retrieval-augmented generation,” which names the actual supporting AI component without overstating its analytical role.

R1-7 — Figures must be high-quality with legible labels. I agree; figures were treated as a resubmission priority. Change: Figures 1–6 were regenerated as high-quality raster files (TIFF) with all internal text in English and legible legends and annotations; figure source data remain in the reproducibility package.

R1-8 — Single exchange rate / volatility warning. I agree. Change: Methods §2.2 now states that COP is the analytical currency, that USD equivalents are approximate interpretive aids only, and that applying a single round reference rate (COP 4,000/USD) across 2020–2025 does not account for exchange-rate volatility; values are order-of-magnitude approximations. The conversion table is cited as S1 Table.

R1-9 — Single independent author: external review or reproducibility. I agree that independent verification matters. While the manuscript is single-authored, I strengthened the reproducibility package so that the analytical workflow can be independently inspected and replicated: source-data tables, figure and table source data, scripts, a column dictionary, a file manifest, and a Zenodo DOI, with the code repository openly available. Change: the Data and Code Availability section and relevant Methods text now emphasize auditability, openness, and reproducibility as the mechanisms supporting independent verification, and the single-author limitation is acknowledged explicitly.

------------------------------------------------------------------------

Response to Reviewer #2

R2-1 — Z-score justification and sensitivity/specificity. I agree; this is addressed jointly with R1-2. The Z-score engine is presented as an exploratory prioritization instrument, not a diagnostic classifier; threshold strata are reported (685 / 528 / 303), and the absence of sensitivity/specificity estimation is stated as a limitation, with validation against adjudicated audit outcomes proposed as future work. Change: Methods §2.4, Discussion §4.2, and Limitations were revised accordingly.

R2-2 — Avoid overinterpretation; remain observational. I agree. Change: Results, Discussion, Limitations, and Conclusions now use cautious observational language. Statistical alerts are described as atypical total-value patterns that may support audit prioritization, and the manuscript states explicitly that they cannot establish corruption, causal mechanisms, or unit-price overpricing without additional evidence.

R2-3 — Discrepancies in contract counts. I agree. Change: the manuscript now distinguishes five nested data scopes: (i) the SECOP-II monitored universe — 272,814 contracts (2015 onward); (ii) the recovered local PostgreSQL snapshot — 956,157 total records, of which 165,549 are pharmaceutical (including live post-2025 monitoring rows); (iii) the closed analytical cohort used here — 162,271 contracts (2020–2025); (iv) the value-share concentration subset — 162,151 positive-value contracts (50,355 normalized suppliers); and (v) the Z-score–analyzable subset — 147,020 contracts in categories with ≥10 observations. Earlier provisional figures (e.g., 162,921 contracts spanning 2020–March 2026) reflected a cohort that included partial 2026 records; closing the cohort to complete calendar years 2020–2025 yields 162,271. Methods §2.2 and the Table 1 footnote now state these scopes, and the manuscript, figures, source data, and reproducibility package were audited so that every count derives from a defined scope.

R2-4 — Lack of structured line-item pricing. I agree. Change: Methods §2.4 and the Limitations now state explicitly that the Z-score engine evaluates total contract values within therapeutic categories and cannot determine unit-price inflation or overpricing without structured item-level quantities, unit prices, dosage forms, and comparable specifications.

R2-5 — Blockchain proof of concept is peripheral. I agree. Change: the blockchain section (Methods §2.6, Results §3.5, Discussion §4.3) was shortened and reframed as a secondary technical-feasibility module tested on the Sepolia testnet only; the text states that it does not provide evidence of real-world payment-cycle reduction, realized savings, or implementation readiness.

R2-6 — Conciseness. I agree. Change: repetitive passages were condensed, particularly in the blockchain discussion, the interpretive-scope statements, the exchange-rate caveats, and the Limitations, while preserving the methodological clarifications requested by both reviewers.

------------------------------------------------------------------------

Summary of Major Revisions

1. Single closed 2020–2025 cohort (162,271 contracts) with a five-scope count reconciliation.

2. Z-score reframed as exploratory audit prioritization; threshold strata 685 / 528 / 303 over 147,020 analyzed contracts.

3. Explicit acknowledgment that sensitivity/specificity require an adjudicated reference set.

4. HHI added (119.26 on the 0–10,000 scale).

5. SISMED 2017–2019 mismatch and absence of line-item pricing emphasized in Methods and Limitations.

6. Causal/accusatory language removed throughout.

7. Blockchain shortened and framed as a secondary proof of concept.

8. Currency convention clarified with an exchange-rate volatility caveat.

9. Ethics statement expanded in Methods.

10. Figures 1–6 regenerated as high-quality English-language files.

11. Supporting Information section and in-text citations added.

12. Reproducibility package, Zenodo DOI, and open code emphasized for independent verification.

Attachment Submitted filename: Response_to_Reviewers.docx

# Peer review: Decision Letter 1

**DOI:** 10.1371/journal.pone.0350967.r003

**Authors:** Xin Sun

17 Jul 2026

Dear Dr. Soto,

Thank you for submitting your manuscript to PLOS ONE. After careful consideration, we feel that it has merit but does not fully meet PLOS ONE’s publication criteria as it currently stands. Therefore, we invite you to submit a revised version of the manuscript that addresses the points raised during the review process.

Your revision have been assessed by the two previous reviewers and they have addressed several concerns. Please read carefully and address them one by one in your next revision.

Please submit your revised manuscript by Aug 31 2026 11:59PM. If you will need more time than this to complete your revisions, please reply to this message or contact the journal office at plosone@plos.org. When you're ready to submit your revision, log on to https://www.editorialmanager.com/pone/ and select the 'Submissions Needing Revision' folder to locate your manuscript file.

- A letter that responds to each point raised by the academic editor and reviewer(s). You should upload this letter as a separate file labeled 'Response to Reviewers'.
- A marked-up copy of your manuscript that highlights changes made to the original version. You should upload this as a separate file labeled 'Revised Manuscript with Track Changes'.
- An unmarked version of your revised paper without tracked changes. You should upload this as a separate file labeled 'Manuscript'.

If you would like to make changes to your financial disclosure, please include your updated statement in your cover letter. Guidelines for resubmitting your figure files are available below the reviewer comments at the end of this letter.

If applicable, we recommend that you deposit your laboratory protocols in protocols.io to enhance the reproducibility of your results. Protocols.io assigns your protocol its own identifier (DOI) so that it can be cited independently in the future. For instructions see: https://journals.plos.org/plosone/s/submission-guidelines#loc-laboratory-protocols. Additionally, PLOS ONE offers an option for publishing peer-reviewed Lab Protocol articles, which describe protocols hosted on protocols.io. Read more information on sharing protocols at https://plos.org/protocols?utm_medium=editorial-email&utm_source=authorletters&utm_campaign=protocols.

As the corresponding author, your ORCID iD is verified in the submission system and will appear in the published article. PLOS supports the use of ORCID, and we encourage all coauthors to register for an ORCID iD and use it as well. Please encourage your coauthors to verify their ORCID iD within the submission system before final acceptance, as unverified ORCID iDs will not appear in the published article. *Only*  the individual author can complete the verification step; PLOS staff *cannot*  verify ORCID iDs on behalf of authors.

We look forward to receiving your revised manuscript.

Kind regards,

Xin Sun, PhD

Staff Editor

PLOS One

Journal Requirements:

If the reviewer comments include a recommendation to cite specific previously published works, please review and evaluate these publications to determine whether they are relevant and should be cited. There is no requirement to cite these works unless the editor has indicated otherwise.

[Note: HTML markup is below. Please do not edit.]

Reviewers' comments:

Reviewer's Responses to Questions

**Comments to the Author**

Reviewer #1: (No Response)

Reviewer #2: (No Response)

**********

2. Is the manuscript technically sound, and do the data support the conclusions?

Reviewer #1: Partly

Reviewer #2: Yes

**********

3. Has the statistical analysis been performed appropriately and rigorously?

Reviewer #1: Yes

Reviewer #2: Yes

**********

4. Have the authors made all data underlying the findings in their manuscript fully available?

The PLOS Data policy

Reviewer #1: Yes

Reviewer #2: Yes

**********

5. Is the manuscript presented in an intelligible fashion and written in standard English?

Reviewer #1: Yes

Reviewer #2: Yes

**********

Reviewer #1: The main revisions have been made to the text, but a few minor clarifications remain:

1. In Section 2.6, regarding the financial rate of 2% per month - at least one reference to a source justifying this figure in the context of the Colombian public procurement market must be added.

2. In the text (Sections 3.4 and 5), the concentration index is listed as 85.87%, while the legend for Fig. 4 states: “Top 3% = 88.9%”

3. Table 1 states: “Unique pharmaceutical suppliers in the closed cohort: 50,460”, section 3.4 states: “showed 50,460 unique supplier names. For value-share concentration metrics, 162,151 positive-value contracts and 50,355 suppliers with non-empty normalized labels were included.” In other words, the same paragraph mentions both 50,460 and 50,355 suppliers without explaining the transition between these numbers.

4. Please explain why, out of the 22,033 indexed contracts for 2025 (Section 3.3), only 6,792 were included in the Z-score engine. Is this due to limitations in the data available at the time of upload, the specifics of categorical filtering, or some other reason?

5. It is recommended to add 1–2 sentences to Section 4.1 discussing the limitation of the calculated HHI, since it is defined for the entire national market, while high concentration is also possible in specific groups (oncology, antibiotics, antivirals), but this is not specified by the author.

Reviewer #2: This manuscript presents a reproducible computational framework for monitoring public pharmaceutical procurement in Colombia using openly available administrative data. The topic is relevant and timely, and the study benefits from a large dataset, transparent methodology, and comprehensive data and code availability.

The manuscript is generally well organized and clearly written. The analyses are adequately described, and the conclusions are mostly supported by the reported findings. The authors should be commended for their efforts to ensure transparency and reproducibility.

I have a few suggestions that could further strengthen the manuscript:

1. The anomaly-detection approach is based primarily on Z-score thresholds. While useful for identifying unusual contract values, the limitations of this method, including the potential for false-positive and false-negative findings, should be discussed in greater detail.

2. The manuscript appropriately notes that statistical alerts do not constitute evidence of corruption or fraud. This distinction should remain clearly emphasized throughout the discussion and conclusions.

3. The smart-contract component is an interesting addition; however, it remains a proof of concept tested under simulated conditions. Its practical implications should continue to be presented cautiously, avoiding any suggestion of demonstrated real-world effectiveness.

4. Additional information regarding the validation of the keyword-based pharmaceutical filtering strategy would strengthen confidence in the construction of the analytical cohort.

5. Several policy-related interpretations are interesting but somewhat speculative. These statements should be framed as potential implications rather than direct conclusions derived from the data.

**********

what does this mean?). If published, this will include your full peer review and any attached files.

If you choose “no”, your identity will remain anonymous but your review may still be made public.

**Do you want your identity to be public for this peer review?**  For information about this choice, including consent withdrawal, please see our Privacy Policy

Reviewer #1: No

Reviewer #2: No

**********

[NOTE: If reviewer comments were submitted as an attachment file, they will be attached to this email and accessible via the submission site. Please log into your account, locate the manuscript record, and check for the action link "View Attachments". If this link does not appear, there are no attachment files.]

To ensure your figures meet our technical requirements, please review our figure guidelines: https://journals.plos.org/plosone/s/figures

You may also use PLOS’s free figure tool, NAAS, to help you prepare publication quality figures: https://journals.plos.org/plosone/s/figures#loc-tools-for-figure-preparation.

NAAS will assess whether your figures meet our technical requirements by comparing each figure against our figure specifications.

# Author comment: Author response to Decision Letter 2

**DOI:** 10.1371/journal.pone.0350967.r004

21 Jul 2026

Dear Dr. Sun and Editors of PLOS ONE,

Thank you for the opportunity to revise manuscript PONE-D-26-13579R1. I have addressed every remaining clarification from Reviewers #1 and #2. The same point-by-point response is also uploaded as the separate file “Response to Reviewers.”

I verified the R1 package on file in Editorial Manager (PONE-D-26-13579_R1). That version matches the intermediate counts cited by the reviewers (for example 50,460 / 50,355 suppliers, 85.87% top-3% concentration, 22,033 / 6,792 contracts for 2025, HHI 119.26, and Z-score denominator 147,020). The present revision retains every clarification below and additionally incorporates the final author-approved contract-level veterinary correction (dated 2026-07-11; previously disclosed to the editor): 161,830 contracts; 50,225 normalized suppliers; top-3% concentration 85.83%; HHI 120.99; and for 2025, 21,955 indexed contracts of which 6,729 enter the Z-score engine. All tables, figures, and reproducibility artifacts are aligned to these corrected figures.

RESPONSE TO REVIEWER #1

R1-1 — Source justifying the 2% per month financial rate (§2.6).

I agree. Change: Methods §2.6 now frames the 2% monthly rate as a conservative upper-bound scenario parameter (approximately 26.8% effective annual), not as an empirically measured average sector rate. The quantitative anchor is the upper range of Colombian legal interest ceilings derived from the interés bancario corriente certified by the Superintendencia Financiera de Colombia, where maximum remuneratory or default interest equals 1.5 times that certified rate (usury ceiling) (refs 17–18). Commercial or rediscount lines can be lower. As contextual motivation for treating payment delay as financially costly—without claiming that AFIDRO measures a 2% monthly rate—we cite the AFIDRO/Sectorial receivables report (ref 19). Results §3.5 use the same “upper-bound scenario parameter” wording.

R1-2 — Concentration 85.87% in text versus a conflicting Fig. 4 annotation (“Top 3% = 88.9%”).

I agree that text and Figure 4 must report the same concentration value. In the R1 Editorial Manager package, §3.4 reported 85.87% while the Abstract/figure annotation used a rounded 85.9%; the reviewer correctly flagged that the figure presentation was not acceptably synchronized with the text. Change: after the contract-level veterinary correction, text (§3.4, §5), source data, and Figure 4 are fully reconciled: the top 3% of suppliers concentrate 85.83% of contracted value, and the figure annotation reads “Top 3% = 85.8%” (50,225 active suppliers; HHI 120.99).

R1-3 — 50,460 versus 50,355 suppliers without explaining the transition.

I agree that supplier scopes must be explicit. Change: Table 1 and §3.4 now use a single concentration denominator—50,225 unique non-empty normalized supplier labels among 161,710 positive-value contracts—and §3.4 states that zero-value or blank-label rows are outside that denominator. The intermediate counts cited by the reviewer correspond to the pre-correction revision and are superseded by the corrected figures above.

R1-4 — Why only 6,792 of 22,033 indexed 2025 contracts entered the Z-score engine.

I agree this required an explicit funnel explanation. The cause is categorical filtering, not incomplete data at upload: the contracts are present in the closed cohort but are ineligible for within-category Z-scores. Change: Methods §2.4 now states that contracts labeled NO_ESPECIFICADO are excluded because they lack a stable within-category comparator, and that named categories with fewer than 10 contracts are also excluded. Results §3.1 list NO_ESPECIFICADO in the therapeutic-category distribution (15,226 contracts; COP 2.63 trillion; 15.5% of corrected-cohort value). Results §3.3 report the corrected 2025 funnel: 21,955 indexed → 6,729 analyzed (30.7%), because all 15,226 NO_ESPECIFICADO contracts fall in 2025 (21,955 − 6,729 = 15,226). Those unspecified contracts account for about 48% of 2025 contracted value, so the 1.31% alert rate describes only the named-category slice and must not be read as an anomaly rate for all 2025 pharmaceutical procurement. Coverage for 2020–2024 remains near-complete under the same rules (aggregate gap of 10 contracts in categories with n<10).

R1-5 — Limitation of national HHI (§4.1).

I agree. Change: Section 4.1 now notes that the national HHI may understate concentration within specific therapeutic groups (for example oncology, antibiotics, or antivirals) and that category-specific HHI values were not estimated. It further clarifies that, under conventional thresholds, HHI = 120.99 would read as unconcentrated at the national aggregate level, yet that interpretation can coexist with the observed top-3% cumulative share of 85.83% when a large long-tail supplier base dilutes the index; subgroup HHI estimation is left for future work.

RESPONSE TO REVIEWER #2

R2-1 — Discuss Z-score limitations, including false positives and false negatives, in greater detail.

I agree. Change: Section 4.2 and the Limitations now expand this point: without an adjudicated audit gold standard, sensitivity and specificity cannot be estimated; the exploratory |Z| ≥ 1.5 threshold favors prioritization recall and can increase false positives; false negatives remain possible under category heterogeneity, NO_ESPECIFICADO exclusion, or small category sizes.

R2-2 — Keep clear that statistical alerts are not evidence of corruption or fraud.

I agree. Change: this distinction is reinforced in Results §3.3, Discussion/Limitations, Abstract Conclusions, and Conclusions §5, which state explicitly that alerts are exploratory prioritization signals and do not constitute evidence of corruption or fraud.

R2-3 — Present the smart-contract component cautiously as a simulated proof of concept.

I agree. Change: the manuscript continues to frame the Sepolia module as a secondary technical-feasibility proof of concept under simulated conditions only. Abstract Results no longer foreground illustrative COP savings figures; they state only that predefined digital state transitions were executed under testnet conditions and that the module provides no evidence of real-world payment-cycle reduction, realized savings, or institutional deployability. Abstract Conclusions and §5 retain the same caution. Detailed scenario arithmetic, where retained, remains confined to Methods §2.6 / Results §3.5 as an explicitly non-empirical illustration.

R2-4 — Additional information on validation of the keyword-based pharmaceutical filter.

I agree. Change: Methods §2.2 now describes the 19-keyword screen as high-recall rather than gold-standard; explains false-positive/false-negative pathways; and reports the contract-level quality-control review of 519 candidates (441 excluded; 27 human-health retained; 51 ambiguous retained), while stating that no independently adjudicated external reference set was available for full taxonomy sensitivity/specificity. The Limitations retain the call for dedicated taxonomy validation.

R2-5 — Frame policy-related statements as potential implications rather than direct conclusions.

I agree. Change: Section 4.6 is retitled “Potential implications for public policy” and uses cautious language (“may inform,” “potential institutional concern”). Conclusions and the Abstract Conclusions retain descriptive findings and avoid prescribing reforms.

SUMMARY OF MAJOR REVISIONS (R2)

1. Justified the 2%/month financing-cost assumption as an upper-bound scenario parameter anchored to Colombian Superintendencia Financiera / usury ceilings (refs 17–18), with AFIDRO receivables context (ref 19).

2. Reconciled concentration reporting and Figure 4 at 85.83% / 85.8% with 50,225 suppliers.

3. Added an explicit supplier-scope bridge for the concentration denominator (§3.4; Table 1).

4. Documented the 2025 Z-score funnel (NO_ESPECIFICADO exclusion; 21,955 → 6,729), including COP 2.63 trillion / ~48% of 2025 value and a cautious reading of the 1.31% alert rate.

5. Strengthened the national-HHI limitation regarding therapeutic subgroups and the coexistence of low aggregate HHI with high top-3% cumulative share (§4.1).

6. Expanded Z-score false-positive/false-negative discussion and reinforced that alerts ≠ corruption/fraud.

7. Strengthened keyword-filter validation disclosure (§2.2) and reframed policy text as potential implications.

Sincerely,

Andrés Soto

Corresponding author

ORCID: 0009-0004-8001-5372

Attachment Submitted filename: Response_to_Reviewers_auresp_2.docx

# Peer review: Decision Letter 2

**DOI:** 10.1371/journal.pone.0350967.r005

**Authors:** Patrick Goymer

20 Aug 2026

Computational surveillance of Colombian public pharmaceutical procurement using public administrative data: a reproducible analysis of a closed 2020-2025 cohort

PONE-D-26-13579R2

Dear Dr. Soto,

We’re pleased to inform you that your manuscript has been judged scientifically suitable for publication and will be formally accepted for publication once it meets all outstanding technical requirements.

Within one week, you’ll receive an e-mail detailing the required amendments. When these have been addressed, you’ll receive a formal acceptance letter and your manuscript will be scheduled for publication.

An invoice will be generated when your article is formally accepted. Please note, if your institution has a publishing partnership with PLOS and your article meets the relevant criteria, all or part of your publication costs will be covered. Please make sure your user information is up-to-date by logging into Editorial Manager at Editorial Manager® and clicking the ‘Update My Information' link at the top of the page. For questions related to billing, please contact billing support.

If your institution or institutions have a press office, please notify them about your upcoming paper to help maximize its impact. If they’ll be preparing press materials, please inform our press team as soon as possible -- no later than 48 hours after receiving the formal acceptance. Your manuscript will remain under strict press embargo until 2 pm Eastern Time on the date of publication. For more information, please contact onepress@plos.org.

Kind regards,

Patrick Goymer

Staff Editor

PLOS One

Additional Editor Comments (optional):

Reviewers' comments:

Reviewer's Responses to Questions

**Comments to the Author**

Reviewer #1: (No Response)

Reviewer #2: All comments have been addressed

**********

2. Is the manuscript technically sound, and do the data support the conclusions?

Reviewer #1: Yes

Reviewer #2: Yes

**********

3. Has the statistical analysis been performed appropriately and rigorously?

Reviewer #1: Yes

Reviewer #2: Yes

**********

4. Have the authors made all data underlying the findings in their manuscript fully available?

The PLOS Data policy

Reviewer #1: Yes

Reviewer #2: Yes

**********

5. Is the manuscript presented in an intelligible fashion and written in standard English?

Reviewer #1: Yes

Reviewer #2: Yes

**********

Reviewer #1: This version of the manuscript has several strengths, including the scope of the data, the transparency of the methodology, and the honest acknowledgment of the study’s limitations. The article represents a substantial contribution to the field of digital infrastructure for government pharmaceutical procurement and may be accepted for publication.

Reviewer #2: I appreciate your careful and thorough revision of the manuscript. The revised version addresses the methodological, statistical, and interpretative concerns raised during the previous rounds of review. In particular, the clarification of the analytical cohort, reconciliation of previously inconsistent counts, expanded discussion of the limitations of the Z-score approach, cautious interpretation of statistical alerts, and improved description of the blockchain component as a technical proof of concept have substantially strengthened the manuscript.

I also appreciate the enhanced transparency achieved through the public availability of the reproducibility package, analytical datasets, source code, and documentation.

The conclusions are now appropriately aligned with the presented evidence, and the limitations are discussed in a balanced manner. Overall, I believe the manuscript is technically sound, clearly written, and suitable for publication in PLOS ONE.

**********

what does this mean?). If published, this will include your full peer review and any attached files.

If you choose “no”, your identity will remain anonymous but your review may still be made public.

**Do you want your identity to be public for this peer review?**  For information about this choice, including consent withdrawal, please see our Privacy Policy

Reviewer #1: No

Reviewer #2: No

**********

# Editor report: Acceptance letter

**DOI:** 10.1371/journal.pone.0350967.r006

**Authors:** Patrick Goymer

PONE-D-26-13579R2

PLOS One

Dear Dr. Soto,

I'm pleased to inform you that your manuscript has been deemed suitable for publication in PLOS One. Congratulations! Your manuscript is now being handed over to our production team.

At this stage, our production department will prepare your paper for publication. This includes ensuring the following:

* All references, tables, and figures are properly cited

* All relevant supporting information is included in the manuscript submission,

* There are no issues that prevent the paper from being properly typeset

You will receive further instructions from the production team, including instructions on how to review your proof when it is ready. Please keep in mind that we are working through a large volume of accepted articles, so please give us a few days to review your paper and let you know the next and final steps.

Lastly, if your institution or institutions have a press office, please let them know about your upcoming paper now to help maximize its impact. If they'll be preparing press materials, please inform our press team within the next 48 hours. Your manuscript will remain under strict press embargo until 2 pm Eastern Time on the date of publication. For more information, please contact onepress@plos.org.

You will receive an invoice from PLOS for your publication fee after your manuscript has reached the completed accept phase. If you receive an email requesting payment before acceptance or for any other service, this may be a phishing scheme. Learn how to identify phishing emails and protect your accounts at https://explore.plos.org/phishing.

If we can help with anything else, please email us at customercare@plos.org.

Thank you for submitting your work to PLOS One and supporting open access.

Kind regards,

PLOS One Editorial Office Staff

on behalf of

Dr Patrick Goymer

Staff Editor

PLOS One
